# Association of prognostic nutritional index level and diabetes status with the prognosis of coronary artery disease: a cohort study

**DOI:** 10.1186/s13098-023-01019-8

**Published:** 2023-03-25

**Authors:** Tianyu Li, Deshan Yuan, Peizhi Wang, Guyu Zeng, Sida Jia, Ce Zhang, Pei Zhu, Ying Song, Xiaofang Tang, Runlin Gao, Bo Xu, Jinqing Yuan

**Affiliations:** 1 National Clinical Research Center for Cardiovascular Diseases, State Key Laboratory of Cardiovascular Disease, Fuwai Hospital, National Center for Cardiovascular Diseases, Chinese Academy of Medical Sciences and Peking Union Medical College, 167, North Lishi Road, Xicheng District, Beijing, 100037 China; 2 Department of Cardiology, Fuwai Hospital, National Center for Cardiovascular Diseases, Chinese Academy of Medical Sciences and Peking Union Medical College, 167, North Lishi Road, Xicheng District, Beijing, 100037 China; 3 Catheterization Laboratories, Fuwai Hospital, National Center for Cardiovascular Diseases, Chinese Academy of Medical Sciences and Peking Union Medical College, 167, North Lishi Road, Xicheng District, Beijing, 100037 China

**Keywords:** Nutritional status, Inflammation, Diabetes mellitus, Ischemic heart disease

## Abstract

**Background:**

Malnutrition and inflammation are associated with adverse clinical outcomes in patients with diabetes or coronary artery disease (CAD). Prognostic nutritional index (PNI) is a comprehensive and simple indicator reflecting nutritional condition and immunological status. Whether there is a crosstalk between nutritional-immunological status and diabetes status for the impact on the prognosis of coronary artery disease (CAD) is unclear.

**Methods:**

A total of 9429 consecutive CAD patients undergoing percutaneous coronary intervention were grouped by diabetes status [diabetes (DM) and non-diabetes (non-DM)] and preprocedural PNI level [high PNI (H-PNI) and low PNI (L-PNI)] categorized by the statistically optimal cut-off value of 48.49. The primary endpoint was all-cause death.

**Results:**

During a median follow-up of 5.1 years (interquartile range: 5.0–5.1 years), 366 patients died. Compared with the non-DM/H-PNI group, the DM/L-PNI group yielded the highest risk of all-cause death (adjusted hazard ratio: 2.65, 95% confidence interval: 1.97–3.56, p < 0.001), followed by the non-DM/L-PNI group (adjusted hazard ratio: 1.44, 95% confidence interval: 1.05–1.98, p = 0.026), while DM/H-PNI was not associated with the risk of all-cause death. The negative effect of L-PNI on all-cause death was significantly stronger in diabetic patients than in nondiabetic patients (p for interaction = 0.037). Preprocedural PNI category significantly improved the Global Registry of Acute Coronary Events (GRACE) risk score for predicting all-cause death in patients with acute coronary syndrome, especially in those with diabetes.

**Conclusions:**

CAD patients with diabetes and L-PNI experienced the worst prognosis. The presence of diabetes amplifies the negative effect of L-PNI on all-cause death. Poor nutritional-immunological status outweighs diabetes in increasing the risk of all-cause death in CAD patients. Preprocedural PNI can serve as an assessment tool for nutritional and inflammatory risk and an independent prognostic factor in CAD patients, especially in those with diabetes.

**Supplementary Information:**

The online version contains supplementary material available at 10.1186/s13098-023-01019-8.

## Backgroud

Malnutrition, manifested as altered body composition and diminished biological function, is not rare in patients with coronary artery disease (CAD) and has been reported to be associated with adverse clinical outcomes [1]. Inflammation has been recognized as a key mediator in the negative impact of malnutrition on the prognosis of cardiovascular disease [2]. Prognostic Nutritional Index (PNI) was first introduced by Buzby et al. in the context of gastrointestinal surgery [3] and modified by Onodera et al. [4]. Calculated from serum albumin levels and absolute lymphocyte counts, this simple and comprehensive index reflects not only protein stores but also the immunological status. Its prognostic value has been examined in malignancy [5], autoimmune disease [6], and heart failure [7–12] and has been reported in several small-scale studies for patients with acute coronary syndrome (ACS) or stable CAD [13–16].

Diabetes is a common cardiovascular risk factor and has been reported to be associated with increased risk of malnutrition [17]. Both malnutrition and diabetes affect systemic metabolism and exacerbate inflammation, driving the development of CAD. However, noModification of Diet in Renal Disease studies have examined how diabetes and coexisting malnutrition affect the prognosis of CAD. Only one study so far has reported the prevalence and prognostic value of malnutrition in CAD patients accompanied by diabetes [18]. Accordingly, this study aimed to investigate the joint effect and interaction between nutritional-immunological status assessed by PNI and diabetes status on the prognosis of the overall CAD population.

## Methods

### Study design, setting, and participants

From January 2013 to December 2013, the cohort study prospectively recruited 10,724 consecutive patients undergoing percutaneous coronary intervention (PCI) at Fuwai Hospital, National Center for Cardiovascular Diseases, Chinese Academy of Medical Sciences, Beijing, China. PCI was performed by experienced interventional cardiologists blinded to the study protocol. Details on catheterization procedures and periprocedural medication were in line with contemporaneous practice guidelines in China. At discharge, all patients without documented contraindications were prescribed statins and dual anti-platelet therapy with aspirin plus clopidogrel. Other cardiovascular medications, such as β-blockers, angiotensin-converting enzyme inhibitors, or angiotensin-receptor blockers, were prescribed according to patients' conditions and contemporaneous guidelines. Baseline and angiography data were extracted from the electronic medical record. Patients were followed up since the date of PCI. Follow-up and outcome information was obtained through clinic visits or telephone interviews by an independent group of clinical research coordinators at one, six, 12, 24 months, and 5 years after discharge. Investigator training and telephone recording were conducted to achieve high-quality results. Endpoint events were adjudicated by two independent cardiologists, and disagreement was resolved by consensus. This study complied with the Declaration of Helsinki. The Ethics Committee of Fuwai Hospital, National Center for Cardiovascular Diseases, approved the study protocol before enrolment (No. 2013–449). All participants provided written informed consent before intervention.

This post hoc analysis investigated the joint effect and interaction between PNI level and diabetes status on 5 year outcomes for CAD patients after PCI. Exclusion criteria were age less than 18 years, unsuccessful PCI, bare-metal stent implantation, end-stage liver or renal disease, systemic inflammatory disease, and missing preprocedural serum albumin and absolute lymphocyte counts data. Participants entering the final analysis were stratified by diabetes status (diabetes [DM] and non-diabetes [non-DM]) and further categorized by the optimal cut-off value of preprocedural PNI (high PNI [H-PNI] and low PNI [L-PNI]) into four groups.

### Blood sampling and laboratory testing

Preprocedural blood samples were collected after emergency admission for unstable patients and after fasting for at least 12 h for stable patients. Postprocedural blood samples were collected within 24 h after PCI. Fasting blood glucose was assayed by an enzymatic hexokinase method. Glycated hemoglobin was assayed using a Tosoh Automated Glycohemoglobin Analyzer (HLC-723G8, Tokyo, Japan). Blood cell counts were measured by an automated blood cell counter. Serum albumin was measured using an automated chemistry analyzer (AU5400, Olympus, Japan) by the bromocresol green dye method. PNI was calculated as 10 × serum albumin (g/L) + 5 × absolute lymphocyte counts (10^9^/L). Estimated glomerular filtration rate was calculated with the modified Modification of Diet in Renal Disease equation [19].

### Outcomes and covariables

The primary endpoint was all-cause death. Secondary endpoints included cardiac death, non-fatal myocardial infarction (MI), non-fatal stroke, unplanned revascularization, and major adverse cardiovascular and cerebrovascular events. All deaths were considered cardiac unless an unequivocal non-cardiac cause could be established. MI was diagnosed based on the Third Universal Definition of Myocardial Infarction. Strokes included ischemic stroke, hemorrhagic stroke, and transient ischemic attack. Unplanned revascularization was defined as repeated coronary artery bypass grafting or PCI of any vessel driven by ischemic symptoms and events.

Body mass index ≥ 25 kg/m^2^ was considered obese based on the World Health Organization standard for Asian populations [20]. Diabetes was defined as fasting blood glucose ≥ 7.0 mmol/L, glycated hemoglobin ≥ 6.5%, oral antidiabetic medication or insulin use, or self-reported diabetes. Hypertension was defined as systolic blood pressure ≥ 140 mmHg, diastolic blood pressure ≥ 90 mmHg, antihypertensive medication use, or self-reported hypertension. Dyslipidemia was diagnosed when at least one of the following criteria was met: total cholesterol ≥ 6.22 mmol/L, total triglyceride ≥ 2.26 mmol/L, low-density lipoprotein cholesterol ≥ 4.14 mmol/L, high-density lipoprotein cholesterol < 1.04 mmol/L, lipid-lowering medication use, or self-reported dyslipidemia [21].

### Statistical analysis

Preprocedural PNI was categorized by the statistically optimal cut-off value for predicting all-cause death determined by recursive partitioning and log-rank tests. Baseline characteristics were compared using Mann–Whitney U tests, Kruskal–Wallis tests, or χ2 tests as appropriate. Categorical and continuous variables were expressed as numbers (percentages) and median [interquartile range], respectively. Correlation between preprocedural PNI and glycemic measures was assessed using Spearman rank correlation analysis.

Survival curves were plotted using Kaplan–Meier method and compared using log-rank tests. Association of preprocedural PNI category and diabetes status with clinical outcomes was examined using Cox proportional-hazards regression by estimating hazard ratios (HRs) and 95% confidence intervals (CIs). Covariables for adjustment included sex, age, hypertension, chronic obstructive pulmonary disease, previous revascularization, previous MI, previous stroke, high-sensitivity C-reactive protein, estimated glomerular filtration rate, and left ventricular ejection fraction, according to clinical plausibility and significance in univariate analysis. In addition, an inverse probability of treatment weighting analysis based on propensity score was undertaken. The propensity score was calculated by logistic regression with variables related to DM, PNI, and/or the outcomes.

Subgroup analysis for all-cause death was performed according to four variables of interest: age (≥ 65 years versus < 65 years), sex (women versus men), body mass index (≥ 25 kg/m^2^ versus < 25 kg/m^2^), and admission presentation (ACS versus chronic coronary syndrome). In sensitivity analysis for all-cause death, we applied five indexes: (1) preprocedural dichotomous PNI grouped by median; (2) preprocedural continuous PNI; (3) postprocedural PNI categorized by the optimal cut-off value; (4) the change in PNI before and after PCI (ΔPNI); (5) malnutrition defined based on the Global Leadership Initiative on Malnutrition (GLIM) criteria [10, 22]—an etiological criterion of inflammation (high-sensitivity C-reactive protein > 3.0 mg/L) plus any of the following phenotypic criteria: low body mass index (< 18.5 kg/m^2^ if < 70 years, or < 20.0 kg/m^2^ if ≥ 70 years) or reduced muscle mass (free fat mass index < 17.0 kg/m^2^ in men or < 15.0 kg/m^2^ in women). Association of preprocedural continuous PNI and ΔPNI with all-cause death was examined with restricted cubic splines with 4 knots.

The added value of the six indexes beyond the Global Register Acute Coronary Events (GRACE) risk score for the ACS population was evaluated by receiver operating characteristic curves and the decision curve analysis and was compared by the area under the curve (AUC), net reclassification improvement (NRI), and integrated discrimination improvement (IDI).

Statistical analyses were conducted with R version 4.2.0 (R Core Team 2022, Vienna, Austria. www.R-project.org). Figures were created by GraphPad Prism version 9.0.0 (GraphPad Software, San Diego, California, USA, www.graphpad.com). Two-tailed p-values of < 0.05 were considered statistically significant.

## Results

### Study population and baseline characteristics

The study population comprised 10,263 patients, of which 9429 (91.87%) patients with complete 5 year follow-up data were available for the final analysis. The number of participants at each stage is described in Additional file 1: Fig. S1. All baseline characteristics of patients followed up and lost to follow-up were comparable (Additional file 1: Table S1). During a median follow-up of 5.1 years (interquartile range: 5.0–5.1 years), 366 all-cause deaths, 219 cardiac deaths, 551 non-fatal MIs, 345 non-fatal strokes, 1371 unplanned revascularizations, and 2143 major adverse cardiovascular and cerebrovascular events were documented. No correlation was observed between preprocedural PNI and fasting blood glucose or glycated hemoglobin (r < 0.200) (Additional file 1: Table S2).

As shown in Table 1, the median age of the study population was 59 years (interquartile range: 51–66 years), 2163 (22.93%) were women, and 3956 (41.96%) had diabetes. The median value of preprocedural PNI was 52.60 for all participants. When patients were stratified by vital status, absolute lymphocyte counts, serum albumin levels, and PNI were significantly lower in patients who had died than in those still alive. Unsurprisingly, patients who survived to the end of5 year follow-up were younger, had fewer comorbidities (diabetes, hypertension, peripheral artery disease, and chronic obstructive pulmonary disease), were less likely to have a previous history of revascularization, MI and stroke, and had higher estimated glomerular filtration rate and left ventricular ejection fraction. The clinical presentation of CAD, cardiovascular medication use, and angiographic characteristics were well-balanced between the two groups.

**Table 1:** Baseline characteristics stratified by vital status at the end of follow-up

Variable	All participants (n = 9429)	Deceased (n = 366)	Survival (n = 9063)	p
Demographic characteristics				
Sex (Women)	2162 (22.93)	95 (25.96)	1067 (22.81)	0.160
Age, years	59 [51, 66]	66 [58, 73]	58 [51, 65]	< 0.001
≥ 65	2623 (27.82)	203 (55.46)	2420 (26.70)	< 0.001
BMI, kg/m^2^	25.91 [23.88, 27.76]	25.71 [23.40, 27.73]	25.91 [23.94, 27.76]	0.052
≥ 25	5742 (60.90)	213 [58.20]	5529 (61.01)	0.280
Current smoking	5363 (56.88)	210 (57.38)	5153 (56.86)	0.844
Clinical characteristics				
Clinical presentation				0.642
ACS	5583 (59.21)	221 (60.38)	5362 (59.16)	
CCS	3846 (40.79)	145 (39.62)	3701 (40.84)	
Hypertension	6576 (69.74)	291 (79.51)	6285 (69.35)	< 0.001
Dyslipidemia	7121 (75.52)	269 (73.50)	6852 (75.60)	0.358
Diabetes	3956 (41.96)	189 (51.64)	3767 (41.56)	< 0.001
Peripheral artery disease	252 (2.67)	16 (4.37)	236 (2.60)	0.040
COPD	220 (2.33)	24 (6.56)	196 (2.16)	< 0.001
Previous revascularization	2468 (26.17)	134 (36.61)	2334 (25.75)	< 0.001
Previous MI	1826 (19.37)	92 (25.14)	1734 (19.13)	0.004
Previous stroke	990 (10.50)	51 (13.93)	939 (10.36)	0.029
Medication at admission				
Aspirin	9315 (98.79)	359 (90.09)	8956 (98.82)	0.209
Clopidogrel	9412 (99.82)	365 (99.73)	9047 (99.82)	0.576
Statins	9051 (95.99)	351 (95.90)	8700 (95.99)	0.929
β-blockers	8493 (90.07)	323 (88.25)	8170 (90.15)	0.234
ACEIs/ARBs	4929 (52.27)	204 (55.74)	4725 (52.14)	0.176
Preprocedural laboratory tests				
ALC, 10^9^/L	1.87 [1.51, 2.30]	1.76 [1.45, 2.20]	1.87 [1.51, 2.31]	< 0.001
Serum albumin, g/L	42.70 [39.90, 45.90]	41.30 [38.70, 44.60]	42.80 [40.00, 45.90]	< 0.001
PNI	52.60 [49.00, 56.15]	50.98 [46.70, 54.65]	52.65 [49.05, 56.25]	< 0.001
hs-CRP, mg/L	1.60 [0.80, 3.59]	2.08 [1.05, 5.25]	1.58 [0.79, 3.54]	< 0.001
Fasting blood glucose, mmol/L	5.48 [4.93, 6.63]	5.70 [5.04, 7.08]	5.47 [4.93, 6.62]	< 0.001
Glycated hemoglobin, %	6.2 [5.8, 6.9]	6.4 [6.0, 7.3]	6.2 [5.8, 6.9]	0.002
eGFR, ml/min/1.73m^2^	118.11 [102.63, 133.24]	111.64 [89.10, 127.53]	118.27 [10.300, 133.50]	< 0.001
≤ 60	92 (0.98)	16 (4.37)	76 (0.84)	< 0.001
LVEF, %	64 [60, 67]	62 [58, 66]	64 [60, 67]	< 0.001
< 40	102 (1.08)	14 (3.83)	88 (0.97)	< 0.001
Angiographic characteristics				
LM/TVD	412 (4.37)	18 (4.92)	394 (4.35)	0.601
SYNTAX score	10 [6, 17]	10 [5, 17]	10 [6, 17]	0.911
SYNTAX category				0.110
≤ 22	8367 (88.74)	313 (85.52)	8054 (88.87)	
22–32	893 (9.47)	43 (11.75)	850 (9.38)	
≥ 33	169 (1.79)	10 (2.73)	159 (1.75)	
DES implantation	8950 (94.92)	340 (92.90)	8610 (95.00)	0.072

Values are presented as number (%) or median [interquartile range]

*ACEI* angiotensin-converting enzyme inhibitor, *ACS* acute coronary syndrome, *ALC* absolute lymphocyte counts, *ARB* angiotensin-receptor blocker, *BMI* body mass index, *CCS* chronic coronary syndrome, *COPD* chronic obstructive pulmonary disease, *DES* drug-eluting stent, *eGFR* estimated glomerular filtration rate, *hs-CRP* high-sensitivity C-reactive protein, *LM/TVD* left main or three-vessel disease, *LVEF* left ventricular ejection fraction, *MI* myocardial infarction, *PNI* prognostic nutritional index, *SYNTAX* synergy between percutaneous coronary intervention with Taxus and cardiac surgery

The optimal cut-off value of preprocedural PNI for predicting all-cause death was 48.49. Table 2 shows baseline characteristics among four groups stratified by preprocedural PNI category and diabetes status. Patients with L-PNI accounted for 22.08% of all participants, 20.88% of the diabetes population, and 22.95% of the nondiabetic population. The DM/L-PNI group had more women and elderly patients than other groups. Patients in the DM/L-PNI group tended to have more comorbidities and previous adverse events and were more likely to have declined renal and cardiac function. The severity of coronary lesions sequentially increased from the non-DM/H-PNI group to the DM/L-PNI group, reflected by more left main or three-vessel disease and higher Synergy between Percutaneous Coronary Intervention with Taxus and Cardiac Surgery (SYNTAX) score.

**Table 2:** Baseline characteristics stratified by DM status and preprocedural PNI level

Variable	Non-DM/H-PNI (n = 4217)	Non-DM/L-PNI (n = 1256)	DM/H-PNI (n = 3130)	DM/L-PNI (n = 826)	p
Demographic characteristics					
Sex (Women)	835 (19.80)	318 (25.32)	784 (25.05)	225 (27.24)	< 0.001
Age, years	56 [49, 63]	62 [55, 70]	58 [51, 65]	64 [58, 71]	< 0.001
≥ 65	872 (20.68)	528 (42.04)	835 (26.68)	388 (46.97)	< 0.001
BMI, kg/m^2^	25.9 [23.9, 27.8]	25.0 [22.9, 26.8]	26.2 [24.2, 28.3]	25.7 [23.7, 27.7]	< 0.001
≥ 25	2555 (60.59)	619 (49.28)	2088 (66.71)	480 (58.11)	< 0.001
Current smoking	2497 (59.21)	672 (53.50)	1750 (55.91)	444 (53.75)	< 0.001
Clinical characteristics					
Clinical presentation					< 0.001
ACS	1744 (41.36)	424 (33.76)	1409 (45.02)	269 (32.57)	
CCS	2473 (58.64)	832 (66.24)	1721 (54.98)	557 (67.43)	
Hypertension	2793 (66.23)	852 (67.83)	2294 (73.29)	637 (77.12)	< 0.001
Dyslipidemia	3085 (73.16)	861 (68.55)	2526 (80.70)	649 (78.57)	< 0.001
Peripheral artery disease	88 (2.09)	26 (2.07)	105 (3.35)	33 (4.00)	< 0.001
COPD	84 (1.99)	45 (3.58)	63 (2.01)	28 (3.39)	0.001
Previous revascularization	947 (22.46)	311 (24.76)	927 (29.62)	283 (34.26)	< 0.001
Previous MI	772 (18.31)	229 (18.23)	634 (20.26)	191 (23.12)	0.004
Previous stroke	359 (8.51)	131 (10.43)	359 (11.47)	141 (17.07)	< 0.001
Medication at admission					
Aspirin	4174 (98.98)	1230 (97.93)	3097 (98.95)	814 (98.55)	0.018
Clopidogrel	4209 (99.81)	1253 (99.76)	3126 (99.87)	824 (99.76)	0.822
Statins	4060 (96.28)	1222 (97.29)	2985 (95.37)	784 (94.92)	0.007
β-blockers	3761 (89.19)	1114 (88.69)	2872 (91.76)	746 (90.31)	< 0.001
ACEIs/ARBs	2048 (48.57)	622 (49.52)	1772 (56.61)	487 (58.96)	< 0.001
Preprocedural laboratory tests					
ALC, 10^9^/L	2.00 [1.64, 2.38]	1.46 [1.20, 1.71]	2.04 [1.67, 2.49]	1.47 [1.19, 1.74]	< 0.001
Serum albumin, g/L	44.20 [41.70, 46.70]	38.60 [37.1, 40.00]	44.10 [41.50, 46.70]	38.40 [36.70, 40.08]	< 0.001
PNI	54.00 [51.35, 57.00]	46.25 [44.55, 47.45]	54.25 [51.50, 57.33]	46.15 [44.31, 47.45]	< 0.001
hs-CRP, mg/L	1.40 [0.72, 2.99]	1.74 [0.82, 4.69]	1.72 [0.87, 3.54]	2.27 [0.98, 9.15]	< 0.001
Fasting blood glucose, mmol/L	5.13 [4.80, 5.54]	4.95 [4.63, 5.34]	7.04 [5.90, 8.45]	6.74 [5.50, 8.40]	< 0.001
Glycated hemoglobin, %	5.9 [5.7, 6.2]	6.0 [5.7, 6.2]	7.2 [6.7, 8.2]	7.2 [6.6, 8.2]	< 0.001
eGFR, ml/min/1.73m^2^	119.16 [104.99, 133.64]	115.59 [101.22, 132.21]	118.23 [102.19, 133.70]	114.85 [94.54, 131.67]	< 0.001
≤ 60	21 (0.50)	11 (0.88)	29 (0.93)	31 (3.75)	< 0.001
LVEF, %	64 [60, 68]	63 [60, 68]	64 [60, 67]	62 [58, 66]	< 0.001
< 40	35 (0.83)	17 (1.35)	30 (0.96)	20 (2.42)	< 0.001
Angiographic characteristics					
LM/TVD	166 (3.94)	55 (4.38)	140 (4.47)	51 (6.17)	0.038
SYNTAX score	9 [6, 16]	10 [6, 17]	10 [6, 17]	11 [6, 19]	< 0.001
SYNTAX category					< 0.001
≤ 22	3794 (89.97)	1122 (89.33)	2757 (88.08)	694 (84.02)	
22–32	359 (8.51)	111 (8.84)	315 (10.06)	108 (13.08)	
≥ 33	64 (1.52)	23 (1.83)	58 (1.85)	24 (2.91)	
DES implantation	4031 (95.59)	1185 (94.35)	2968 (94.82)	766 (92.74)	0.005

Preprocedural PNI was categorized by the optimal cut-off value for all-cause death of 48.49

Values are presented as number (%) or median [interquartile range]

*ACEI* angiotensin-converting enzyme inhibitor, *ACS* acute coronary syndrome, *ALC* absolute lymphocyte counts, *ARB* angiotensin-receptor blocker, *BMI* body mass index, *CCS* chronic coronary syndrome, *COPD* chronic obstructive pulmonary disease, *DES* drug-eluting stent, DM diabetes, *eGFR* estimated glomerular filtration rate, *H* high, *hs-CRP* high-sensitivity *C*-reactive protein; *L* low *LM/TVD* left main or three-vessel disease, *LVEF* left ventricular ejection fraction, *MI* myocardial infarction, *PNI* prognostic nutritional index, *SYNTAX* synergy between percutaneous coronary intervention with Taxus and cardiac surgery

### Effect of preprocedural PNI category and diabetes status on clinical outcomes

**Fig. 1:**
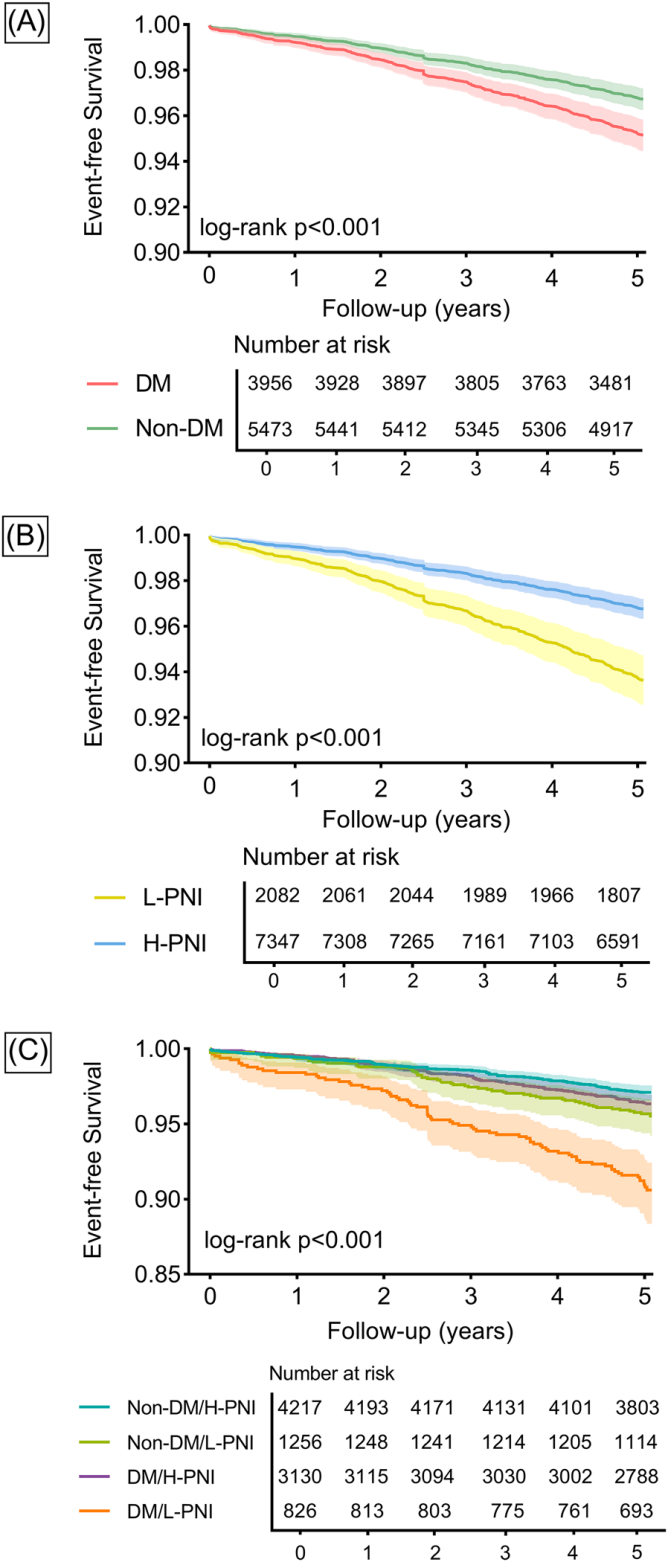
Kaplan–Meier analysis for all-cause death. Survival curves stratified by diabetes status (**A**), PNI level (**B**), and both (**C**). Preprocedural PNI was categorized by the optimal cut-off value for all-cause death of 48.49. *DM* diabetes, *H* high, *L* low, *PNI* prognostic nutritional index.

Kaplan–Meier curves illustrate that patients in the DM/L-PNI group experienced more all-cause deaths than in other groups (log-rank p < 0.001; Fig. 1).

**Fig. 2:**
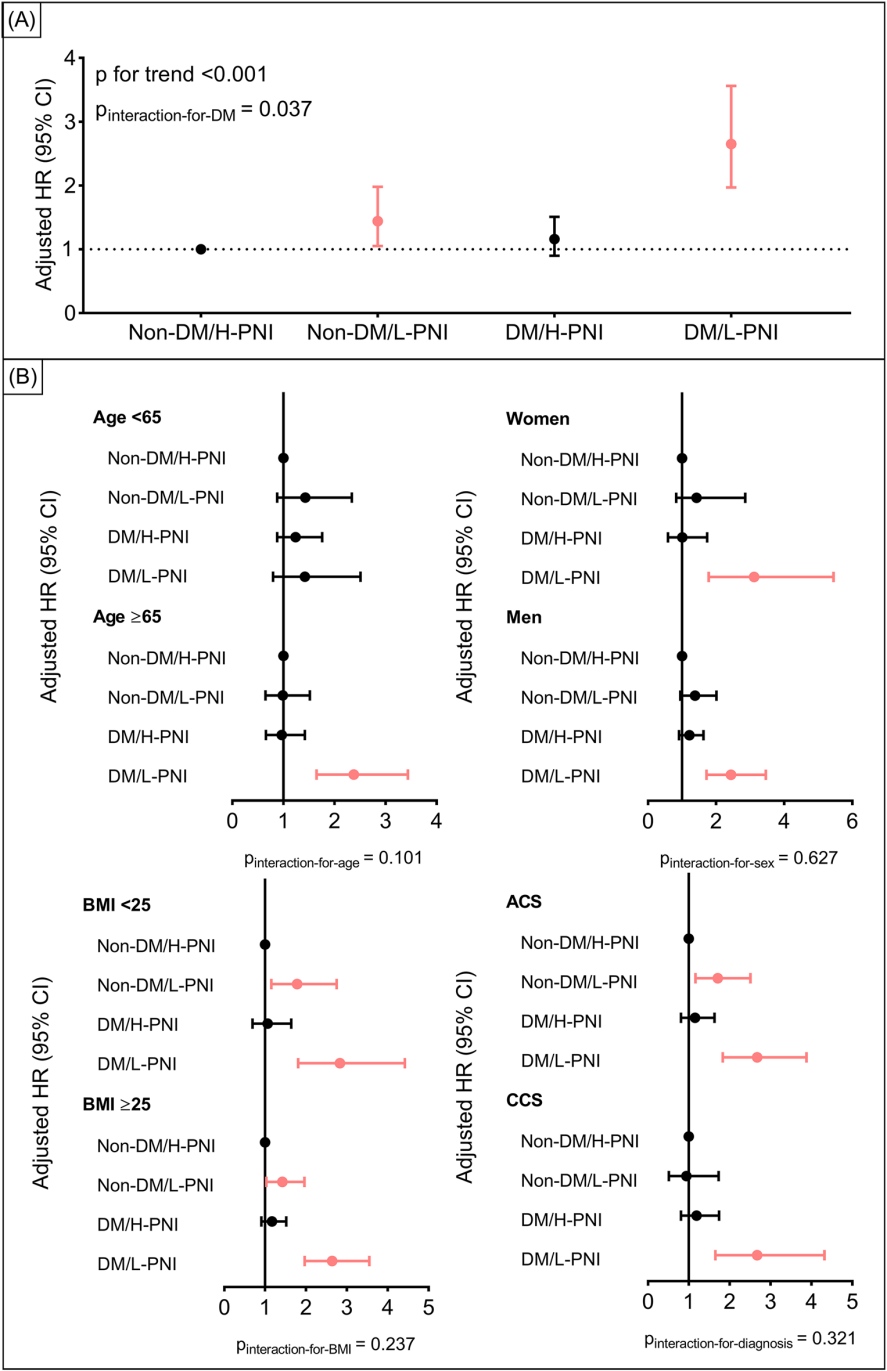
Association of PNI level and diabetes status with all-cause death. Forrest plots for all participants (**A**) and subgroups (**B**). Preprocedural PNI was categorized by the optimal cut-off value for all-cause death of 48.49. Subgroups were defined by age category, sex, BMI category, and admission presentation. *ACS* acute coronary syndrome, *BMI* body mass index, *CCS* chronic coronary syndrome, *CI* confidence interval, *HR* hazard ratio, other abbreviations as in Fig. 1.

Univariate analysis for all-cause death is shown in Additional file 1: Table S3. Analyses before and after adjustment generated consistent results that the DM/L-PNI group yielded the highest risk of all-cause death (adjusted HR: 2.65, 95% CI 1.97–3.56, p < 0.001) compared with the non-DM/H-PNI group, followed by the non-DM/L-PNI group (adjusted HR: 1.44, 95% CI 1.05–1.98, p = 0.026), while DM/H-PNI was not associated with the risk of all-cause death (Table 3). The negative effect of L-PNI on all-cause death was significantly stronger in diabetic patients than in nondiabetic patients (p for interaction = 0.037; Fig. 2). The inverse probability of treatment weighting analysis produced similar results (Additional file 1: Table S4). Baseline characteristics after weighting were shown in Additional file 1: Table S5.

**Table 3:** Associations of DM status and PNI level with clinical outcomes

Outcome	Events/Total	Event rate per 1000 pys	Crude HR (95% CI)	p	Adjusted HR (95% CI)	p
All-cause death	366/9429	7.87	**–**	**–**	**–**	**–**
Non-DM/H-PNI	122/4217	5.83	Reference	**–**	Reference	**–**
Non-DM/L-PNI	55/1256	8.89	1.53 (1.11, 2.10)	0.009	1.44 (1.05, 1.98)	0.026
DM/H-PNI	113/3130	7.32	1.26 (0.97, 1.62)	0.080	1.16 (0.90, 1.51)	0.248
DM/L-PNI	76/826	19.16	3.30 (2.47, 4.39)	< 0.001	2.65 (1.97, 3.56)	< 0.001
p for trend	–	–	< 0.001	–	< 0.001	–
Cardiac death	219/9429	4.71	**–**	**–**	**–**	**–**
Non-DM/H-PNI	69/4217	3.30	Reference	–	Reference	**–**
Non-DM/L-PNI	35/1256	5.66	1.72 (1.14, 2.58)	0.009	1.61 (1.07, 2.43)	0.022
DM/H-PNI	67/3130	4.34	1.32 (0.94, 1.84)	0.107	1.21 (0.86, 1.69)	0.274
DM/L-PNI	48/826	12.10	3.68 (2.54, 5.31)	< 0.001	2.83 (1.94, 4.14)	< 0.001
p for trend	–	–	< 0.001	–	< 0.001	–
Non-fatal MI	551/9429	12.18	**–**	–	**–**	**–**
Non-DM/H-PNI	236/4217	11.58	Reference	–	Reference	**–**
Non-DM/L-PNI	65/1256	10.75	0.93 (0.71, 1.22)	0.600	0.91 (0.69, 1.20)	0.495
DM/H-PNI	199/3130	13.28	1.15 (0.95, 1.39)	0.152	1.08 (0.89, 1.31)	0.423
DM/L-PNI	51/826	13.28	1.14 (0.84, 1.55)	0.386	1.03 (0.76, 1.40)	0.837
p for trend	–	–	0.1358		0.4946	
Non-fatal stroke	345/9429	7.54	**–**	**–**	**–**	–
Non-DM/H-PNI	115/4217	5.57	Reference	**–**	Reference	**–**
Non-DM/L-PNI	59/1256	9.74	1.75 (1.28, 2.39)	0.001	1.68 (1.22, 2.30)	0.001
DM/H-PNI	131/3130	8.66	1.56 (1.21, 2.00)	0.001	1.46 (1.14, 1.88)	0.003
DM/L-PNI	40/826	10.32	1.85 (1.29, 2.66)	0.001	1.63 (1.13, 2.35)	0.009
p for trend	–	–	< 0.001	–	0.001	–
Unplanned revascularization	1371/9429	32.46	**–**	**–**	**–**	**–**
Non-DM/H-PNI	577/4217	30.21	Reference	**–**	Reference	**–**
Non-DM/L-PNI	154/1256	26.98	0.89 (0.75, 1.07)	0.217	0.89 (0.75, 1.07)	0.220
DM/H-PNI	519/3130	37.50	1.23 (1.10, 1.39)	0.001	1.21 (1.07, 1.36)	0.002
DM/L-PNI	121/826	33.81	1.11 (0.91, 1.35)	0.308	1.07 (0.88, 1.31)	0.475
p for trend	–	–	0.003	–	0.011	–
MACCE	2143/9429	52.36	**–**	**–**	**–**	**–**
Non-DM/H-PNI	851/4217	45.71	Reference	**–**	Reference	**–**
Non-DM/L-PNI	269/1256	48.66	1.06 (0.93, 1.22)	0.375	1.04 (0.91, 1.20)	0.543
DM/H-PNI	777/3130	58.17	1.26 (1.55, 1.39)	< 0.001	1.21 (1.10, 1.34)	< 0.001
DM/L-PNI	246/826	71.86	1.55 (1.35, 1.79)	< 0.001	1.42 (1.23, 1.65)	< 0.001
p for trend	–	–	< 0.001	–	< 0.001	–

Preprocedural PNI was categorized by the optimal cut-off value for all-cause death of 48.49. Adjusted for sex, age, hypertension, chronic obstructive pulmonary disease, previous revascularization, previous myocardial infarction, previous stroke, high-sensitivity C-reactive protein, estimated glomerular filtration rate, and left ventricular ejection fraction

*CI* confidence interval, *DM* diabetes, *H* high, *H*R hazard ratio, *L* low, *PNI* prognostic nutritional index, pys person years, *MACCE* major adverse cardiovascular and cerebrovascular events, *MI* myocardial infarction

No significant interaction between subgroups and preprocedural PNI category and diabetes status (all p for interaction > 0.05) was detected. DM/L-PNI remained associated with the highest risk of all-cause death, except in the subgroup aged < 65 years which limited statistical power with only 15 all-cause deaths and 438 individuals (Fig. 2, Additional file 1: Table S6).

Kaplan–Meier curves for secondary endpoints are shown in Additional file 1: SFigs. S2, S3, S4, S5, and S6. The same pattern of the association of preprocedural PNI category and diabetes status with all-cause death was observed for cardiac death. The non-DM/H-PNI group yielded a significantly lower risk of non-fatal stroke than the other three groups. DM/H-PNI was associated with an increased risk of unplanned revascularization. DM/H-PNI and DM/L-PNI were associated with an increased risk of major adverse cardiovascular and cerebrovascular events. No association of the four groups with non-fatal MI was observed.

### Sensitivity analysis

Postprocedural PNI decreased in approximately 85% of patients. Analyses applying preprocedural dichotomous PNI and postprocedural PNI category generated robust results with the main analysis, whereas ΔPNI had no association with all-cause death. Only 471 patients were diagnosed with malnutrition based on the GLIM criteria, and the association with all-cause death remained similar to the main analysis (Additional file 1: Table S7).

**Fig. 3:**
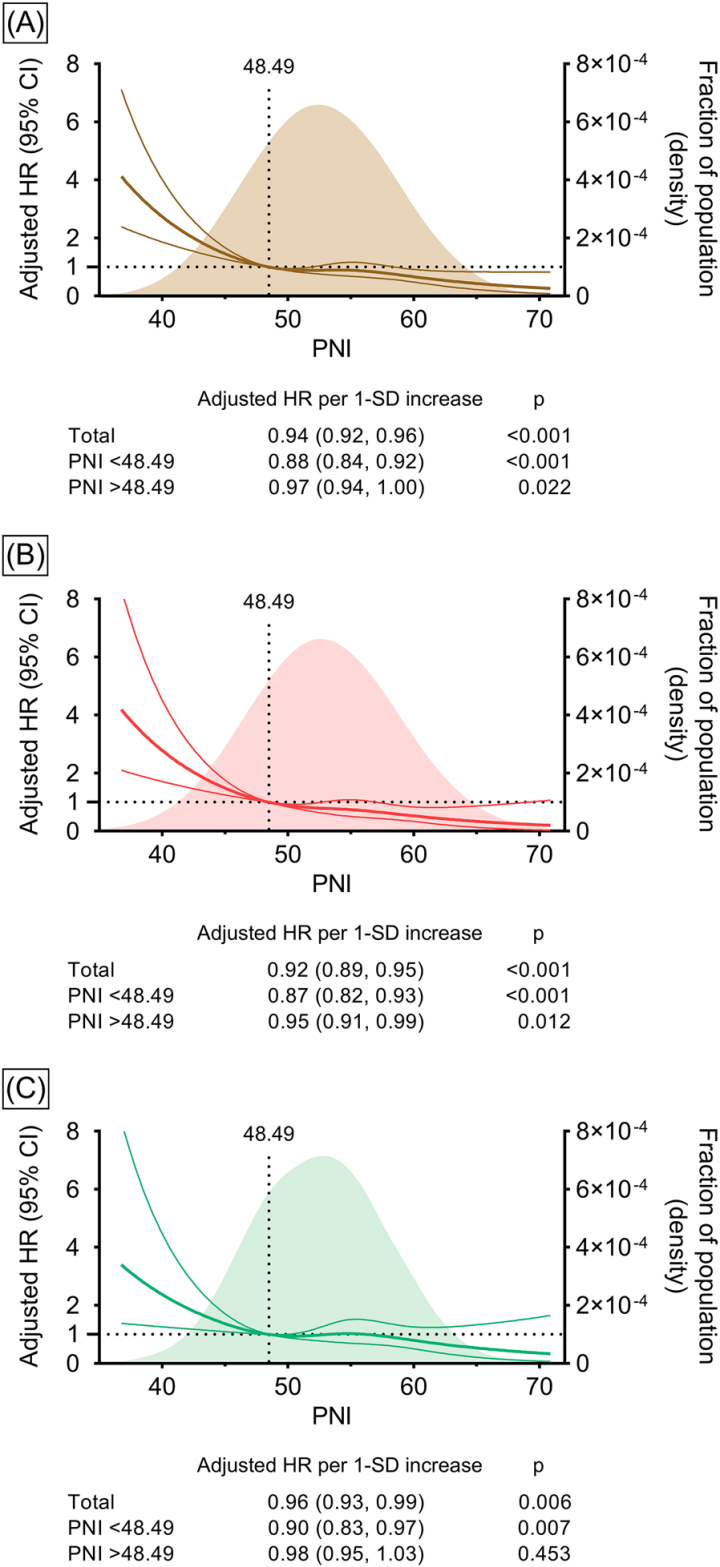
Association of preprocedural continuous PNI with all-cause death. Restricted cubic spline curves for all participants (**A**), diabetic (**B**), and nondiabetic (**C**) patients. A preprocedural PNI level of 48.49 was set as a reference. Adjusted for sex, age, hypertension, chronic obstructive pulmonary disease, previous revascularization, previous myocardial infarction, previous stroke, high-sensitivity C-reactive protein, estimated glomerular filtration rate, and left ventricular ejection fraction. SD, standard deviation; other abbreviations as in Figs. 1, 2.

On a continuous scale, elevated preprocedural PNI was associated with a decreased risk of all-cause death. For a 1-standard deviation increase in PNI, adjusted HRs and 95% Cis were 0.94 (0.92–0.96) in all participants, 0.92 (0.89–0.95) in diabetic patients, and 0.96 (0.93–0.99) in nondiabetic patients. When PNI was below 48.49, the risk of all-cause death decreased sharply with elevating PNI in both diabetic and nondiabetic patients, while a PNI above 48.49 yielded a trend toward a slight but steady reduction in the risk of all-cause death, which was only significant in diabetic patients (Fig. 3).

### Added value of nutritional indexes beyond the GRACE risk score

**Fig. 4:**
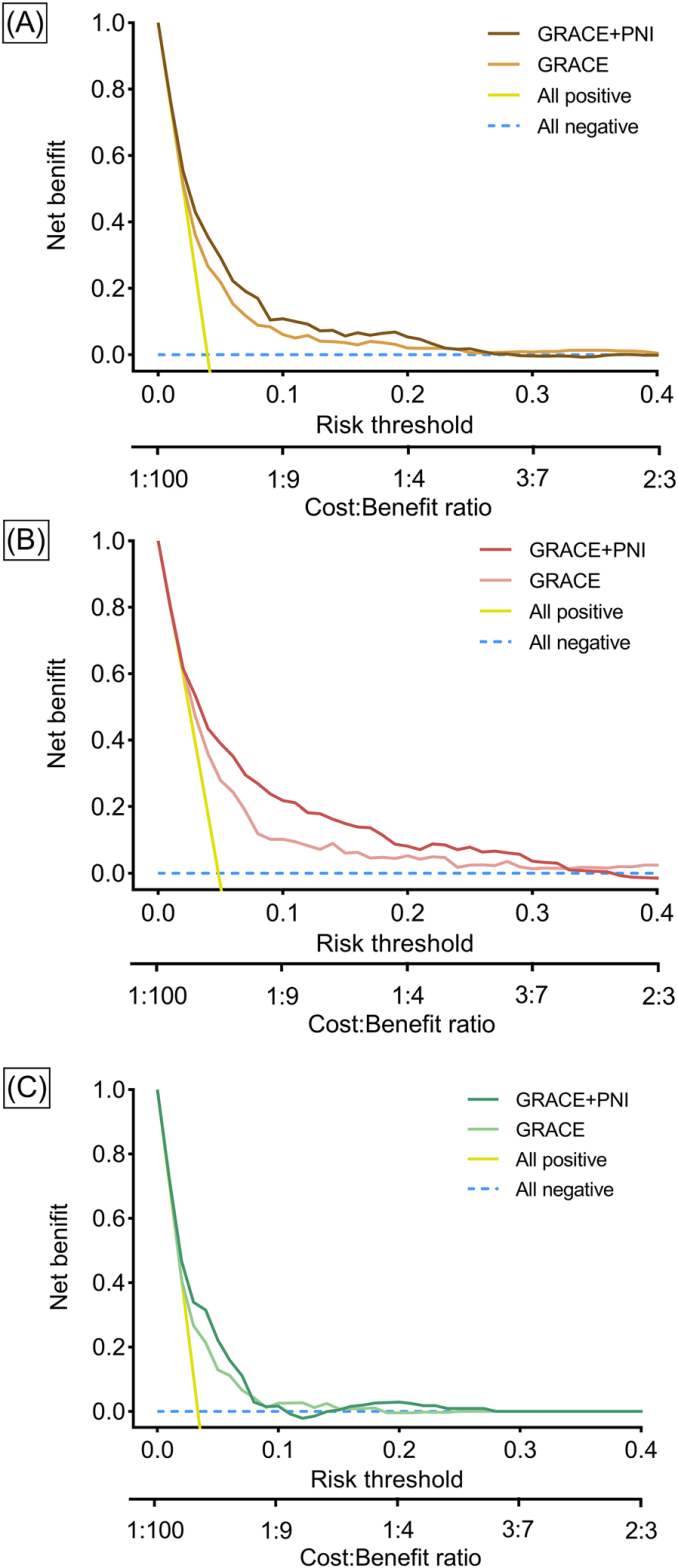
Decision curve analysis for models predicting all-cause death. Decision curves for all participants (**A**), diabetic (**B**), and nondiabetic (**C**) patients. Preprocedural PNI was categorized by the optimal cut-off value for all-cause death of 48.49. GRACE, Global Register Acute Coronary Events; other abbreviations as in Fig. 1.

For the prediction of all-cause death in the entire ACS patients, the addition of preprocedural PNI category significantly improved discrimination (AUC and 95% CI 0.733 [0.698–0.768] vs. 0.688 [0.651–0.725], ΔAUC: 0.045, p < 0.001) and reclassification (NRI: 0.323, 95% CI 0.186–0.466, p < 0.001; IDI: 0.080, 95% CI 0.023–0.137, p = 0.006) of the GRACE risk score (Table 4). The decision curve illustrates that the GRACE + PNI category model outperformed the GRACE risk score, with a higher clinical net benefit within a threshold probability range from 0.05 to 0.25 (Fig. 4A). In diabetic ACS patients, the added value of preprocedural PNI category was more significant, with a higher clinical net benefit within a threshold probability range from 0.05 to 0.30 (Table 4; Fig. 4B). In nondiabetic ACS patients, the addition of preprocedural PNI category also achieved model improvement, whereas the decision curve reveals no clear increase in clinical net benefit (Fig. 4C).

**Table 4:** Model performance after adding nutrition indexes to the GRACE risk score for predicting all-cause death

	AUC (95% CI)	p	NRI (95% CI)	p	IDI (95% CI)	p
All participants						
GRACE	0.688 (0.651, 0.725)	**–**	Reference	**–**	Reference	**–**
GRACE+PNI category^a^	0.733 (0.698, 0.768)	<0.001	0.323 (0.186, 0.466)	<0.001	0.080 (0.023, 0.137)	0.006
GRACE+dichotomous PNI^b^	0.730 (0.695, 0.765)	<0.001	0.221 (− 0.176, 0.350)	0.819	0.088 (0.032, 0.144)	0.002
GRACE+continuous PNI^c^	0.733 (0.698, 0.768)	<0.001	0.102 (− 0.041, 0.262)	0.326	0.094 (0.037, 0.151)	0.001
GRACE+postprocedural PNI^d^	0.731 (0.694, 0.768)	<0.001	0.202 (− 0.203, 0.340)	0.142	0.075 (0.014, 0.136)	0.015
GRACE+ΔPNI^e^	0.694 (0.656, 0.733)	0.119	0.079 (− 0.048, 0.230)	0.449	1x10^-4^ (-0.044, 0.044)	0.998
GRACE+GLIM^f^	0.706 (0.670, 0.743)	0.026	− 0.128 (− 0.326, 0.201)	0.318	0.024 (− 0.027, 0.074)	0.363
Diabetic patients						
GRACE	0.707 (0.657, 0.756)	**–**	Reference	**–**	Reference	**–**
GRACE+PNI category^a^	0.763 (0.713, 0.813)	<0.001	0.414 (0.179, 0.628)	<0.001	0.089 (0.023, 0.154)	0.008
GRACE+dichotomous PNI^b^	0.762 (0.713, 0.811)	<0.001	0.346 (0.129, 0.528)	<0.001	0.091 (0.018, 0.164)	0.015
GRACE+continuous PNI^c^	0.766 (0.718, 0.815)	<0.001	0.228 (0.018, 0.441)	0.019	0.104 (0.031, 0.178)	0.005
GRACE+postprocedural PNI^d^	0.746 (0.692, 0.800)	0.003	0.240 (− 0.236, 0.404)	0.349	0.055 (-0.010, 0.120)	0.097
GRACE+ΔPNI^e^	0.704 (0.652, 0.755)	0.310	0.147 (− 0.067, 0.375)	0.493	− 0.019 (− 0.073, 0.035)	0.495
GRACE+GLIM^f^	0.741 (0.692,0.790)	0.009	− 0.267 (− 0.394, 0.421)	0.288	0.08 (0.005, 0.149)	0.036
Nondiabetic patients						
GRACE	0.662 (0.608, 0.716)	**–**	Reference	**–**	Reference	**–**
GRACE+PNI category^a^	0.716 (0.667, 0.764)	0.001	0.261 (0.118, 0.449)	0.016	0.104 (0.019, 0.188)	0.017
GRACE+dichotomous PNI^b^	0.714 (0.665, 0.762)	0.002	− 0.104 (-0.161, 0.294)	0.543	0.090 (0.006, 0.173)	0.036
GRACE+continuous PNI^c^	0.713 (0.665, 0.762)	0.002	− 0.007 (-0.117, 0.266)	0.607	0.103 (0.020, 0.187)	0.015
GRACE+postprocedural PNI^d^	0.725 (0.674, 0.775)	0.002	− 0.179 (-0.198, 0.369)	0.416	0.045 (− 0.017, 0.160)	0.112
GRACE+ΔPNI^e^	0.675 (0.617, 0.732)	0.133	0.037 (− 0.095, 0.242)	0.562	− 3x10^-4^ (-0.002, 0.002)	0.706
GRACE+GLIM^f^	0.678 (0.626, 0.731)	0.243	− 0.131 (− 0.241, 0.270)	0.352	0.025 (− 0.052, 0.101)	0.527

^a^Preprocedural PNI categorized by the optimal cut-off value for all-cause death of 48.49

^b^Preprocedural PNI grouped by the median

^c^Preprocedural PNI analyzed as a continuous variable

^d^Postprocedural PNI categorized by 48.49

^e^A continuous variable calculated as postprocedural PNI minus preprocedural PNI

^f^Malnutrition defined by the GLIM criteria

*AUC*: area under the curve, *CI* confidence interval, *GLIM* Global Leadership Initiative on Malnutrition, *GRAC*E global register acute coronary events, *IDI* integrated discrimination improvement, *NRI* net reclassification improvement, *PNI* prognostic nutritional index

The addition of preprocedural dichotomous PNI, preprocedural continuous PNI, postprocedural PNI category, and malnutrition defined by the GLIM criteria to the GRACE risk score improved the AUC to varying extents. However, NRI and IDI indicate that these indexes were inferior to preprocedural PNI category. ΔPNI provided no improvement in the GRACE risk score (Table 4).

## Discussion

This study presents the first evaluation of the joint effect and interaction between PNI level and diabetes status on 5 year outcomes after PCI in CAD patients. We found that patients with diabetes and L-PNI experienced the highest risk of all-cause death; the negative effect of L-PNI on all-cause death was significantly stronger in diabetic patients than in nondiabetic patients; the addition of preprocedural PNI category significantly improved model performance and clinical net benefit of the GRACE risk score for predicting all-cause death, especially in diabetic patients. These findings emphasize the prognostic significance of nutritional-immunological status and its interaction with diabetes status for CAD patients.

Previous small-scale studies have reported reduced coronary blood flow [15] and survival rate related to L-PNI in the ACS or stable CAD population [13–16]. This study demonstrated the adverse prognostic significance of low PNI for the overall CAD population. Hypoalbuminemia raises cardiovascular risk mainly related to weakened antioxidant, oncotic pressure-maintaining, and antithrombotic capacities of albumin [24]. In addition, decreased serum albumin indicates underlying inflammation, which provokes the progress of atherosclerosis [25]. Reduced absolute lymphocyte counts indicate impaired immune defenses due to malnutrition [26], reflecting increased susceptibility to infection and inflammation, which translate into atherosclerotic burden [2]. Additionally, different lymphocyte subsets are known to have opposite roles: T helper-1 and B2 cells can induce atherosclerosis, while regulatory T cells and B1 cells have atheroprotective properties [27]. Malnutrition may alter the proportions of lymphocyte subsets, causing an imbalance between proatherogenic and antiatherogenic immune microenvironments [26].

After considering diabetes status, we found that CAD patients accompanied by diabetes and L-PNI experienced the highest risk of all-cause death, the L-PNI-related risk outweighed the diabetes-related risk, while diabetes aggravated the negative impact of L-PNI (Additional file 1: Fig. S7). First, diabetic patients are often in a negative nitrogen balance due to increased protein catabolism and excretion and decreased protein anabolism. This raises the risk of malnutrition, [17] which in turn exacerbates insulin resistance, leading to a vicious cycle that impairs patients’ general conditions. Both diabetes and malnutrition can exacerbate the imbalance between cardioprotective immune response and inflammation, synergistically promoting the progression of CAD, resulting in worse prognosis in patients with combined traits [26, 28, 29]. Second, L-PNI/nondiabetic patients had a higher HR for all-cause death than H-PNI/diabetic patients, which is in line with previous research suggesting that the mortality risk related to malnutrition is higher than that associated with other chronic comorbidities [17], highlighting the value of PNI as a potent and general prognostic indicator. The differential impact of PNI and diabetes on all-cause death may be attributed to the fact that diabetes is typically subjected to active management, whereas subclinical malnutrition often goes undetected and therefore lacks intervention. Last, the adverse prognostic effect of L-PNI was aggravated in the presence of diabetes, which should be explained by the distinct pathophysiological state of diabetic patients. One possible example is that serum albumin might play a role in preventing autophagy; [30] however, the level of autophagy in diabetic heart tissue is significantly increased, [31] thereby amplifying the deleterious impact of hypoalbuminemia.

This study provides a comprehensive analysis of PNI. Except for ΔPNI, preprocedural PNI category, preprocedural dichotomous PNI, preprocedural continuous PNI, and postprocedural PNI category were all significantly associated with all-cause death in CAD patients and improved the AUC of the GRACE risk score. The finding is supported by previous studies [13, 16]. In this study, the GRACE + PNI category model showed the best performance, and only this model achieved significant improvement in both diabetes and nondiabetic patients. Restricted cubic spline for the association of preprocedural continuous PNI with the risk of all-cause death presents an inflection, illustrating that categorizing PNI by a certain cut-off value to identify malnourished patients is clinically realistic. The observed decrease in PNI after PCI may be attributable to the acute stress of catheterization. Therefore, preprocedural PNI is a more appropriate index of nutrition status than postprocedural PNI.

The GLIM has built a global consensus for malnutrition diagnosis with consideration of inflammation. However, the addition of malnutrition defined by the GLIM criteria had limited improvement in the GRACE risk score. This finding can be attributed to two reasons: first, we applied only one etiological criterion and two phenotypic criteria and thus failed to identify all malnourished patients; second, the GLIM still primarily considers body weight, thereby underestimating malnutrition in this study population. Moreover, GLIM criteria involve a multi-step diagnostic approach. In contrast, due to the wide availability of serum albumin and absolute lymphocyte counts, preprocedural PNI is a convenient and potent prognostic factor for CAD patients.

To our knowledge, this large-scale cohort study presents the first evaluation of the prognostic significance of PNI in the overall CAD population, the first investigation of the joint effect and interaction between PNI level and diabetes status on the prognosis of CAD patients, and the most comprehensive analysis for PNI.

This study also has some limitations. First, the observational nature raises concerns about residual confounding. Second, this single-center study was conducted only in Chinese population, which restricts the generalizability of our work. Large-scale studies in different countries and races are needed to determine a universal or race-specific cut-off value of PNI. Third, we did not follow up on nutritional status, which might have changed during the five-year follow-up period. Randomized trials are necessary to evaluate the value of PNI as an indicator of the efficacy of oral nutritional support in improving prognosis of CAD in a context of reduction of inflammatory drivers of both diabetes and CAD.

## Conclusions

CAD patients with diabetes and L-PNI experienced the worst prognosis. The presence of diabetes amplifies the negative effect of status-PNI on all-cause death. Poor nutritional-immunological status outweighs diabetes in increasing the risk of all-cause death in CAD patients. Preprocedural PNI can serve as an assessment tool of nutritional and inflammatory risk and an independent prognostic factor in CAD patients, especially in those with diabetes.

## Supplementary Information

**Additional file1: Table S1.** Baseline characteristics stratified by follow-up status. **Table S2.** Correlation between preprocedural PNI and glycemic measures in all participants and stratified by DM status. **Table S3.** Univariate and multivariate Cox proportional-hazard regression analysis for all-cause death. **Table S4.** Subgroup analysis for all-cause death. **Figure S1.** The study flowchart. **Figure S2.** Kaplan-Meier curves for cardiac death by diabetes status (**A**), PNI level (**B**) and both (**C**). **Figure S3.** Kaplan-Meier curves for non-fatal MI by diabetes status (**A**), PNI level (**B**) and both (**C**). **Figure S4. **Kaplan-Meier curves for non-fatal stroke by diabetes status (**A**), PNI level (**B**) and both (**C**).** Figure S5.** Kaplan-Meier curves for unplanned revascularization by diabetes status (**A**), PNI level (**B**) and both (**C**). **Figure S6.** Kaplan-Meier curves for MACCE by diabetes status (**A**), PNI level (**B**) and both (**C**). **Figure S7.** Central illustration.

## Data Availability

The data that support the findings of this study are available from the Information Center of Fuwai Hospital but restrictions apply to the availability of these data, which were used under license for the current study, and so are not publicly available. Data are however available from the authors upon reasonable request and with permission of the Information Center of Fuwai Hospital.

## Abbreviations

ACS: Acute coronary syndrome
AUC: Area under the curve
CAD: Coronary artery disease
CI: Confidence interval
GLIM: Global leadership initiative on malnutrition
GRACE: Global register acute coronary events
H: High
HR: Hazard ratio
IDI: Integrated discrimination improvement
L: Low
MI: Myocardial infarction
NRI: Net reclassification improvement
PCI: Percutaneous coronary intervention
PNI: Prognostic nutritional index
