# Supplementary material for: Association of prognostic nutritional index level and diabetes status with the prognosis of coronary artery disease: a cohort study
Source: Diabetol Metab Syndr. 2023 Mar 25;15:58. doi: 10.1186/s13098-023-01019-8 (PMC10039549; doi:10.1186/s13098-023-01019-8)
Supplement: Supplementary file 1 — Additional file1: Table S1. Baseline characteristics stratified by follow-up status. Table S2. Correlation between preprocedural PNI and glycemic measures in all participants and stratified by DM status. Table S3. Univariate and multivariate Cox proportional-hazard regression analysis for all-cause death. Table S4. Subgroup analysis for all-cause death. Figure S1. The study flowchart. Figure S2. Kaplan-Meier curves for cardiac death by diabetes status (A), PNI level (B) and both (C). Figure S3. Kaplan-Meier curves for non-fatal MI by diabetes status (A), PNI level (B) and both (C). Figure S4. Kaplan-Meier curves for non-fatal stroke by diabetes status (A), PNI level (B) and both (C). Figure S5. Kaplan-Meier curves for unplanned revascularization by diabetes status (A), PNI level (B) and both (C). Figure S6. Kaplan-Meier curves for MACCE by diabetes status (A), PNI level (B) and both (C). Figure S7. Central illustration. [file 13098_2023_1019_MOESM1_ESM.docx]

**Additional file material for:**

**Association of Prognostic Nutritional Index level and diabetes status with the prognosis of coronary artery disease: a cohort study**

**Additional file 1: Table S1** Baseline characteristics stratified by follow-up status 2

[**Additional file 1: Table S2** Correlation between preprocedural PNI and glycemic measures in all participants and stratified by DM status 4](#_Toc112007683)

[**Additional file 1: Table S3** Univariate and multivariate Cox proportional-hazard regression analysis for all-cause death 5](#_Toc112007684)

[**Additional file 1: Table S4** Subgroup analysis for all-cause death 7](#_Toc112007685)

[**Additional file 1: Table S5** Sensitivity analysis for all-cause death 9](#_Toc112007686)

[**Additional file 1 Figure S1** The study flowchart 11](file:///C:\Users\Tianyu%20Li\Documents\Fuwai\5%20项目\2021-2022\Research_PNI%20(2022.6)\4%20Publication\CD\Additional%20file.docx#_Toc119793879)

**Additional file 1: Figure S2** Kaplan-Meier curves for cardiac death by diabetes status (A), PNI level (B) and both (C) 12

[**Additional file 1: Figure S3** Kaplan-Meier curves for non-fatal MI by diabetes status (A), PNI level (B) and both (C) 13](file:///C:\Users\Tianyu%20Li\Documents\Fuwai\5%20项目\2021-2022\Research_PNI%20(2022.6)\4%20Publication\CD\Additional%20file.docx#_Toc119793881)

[**Additional file 1: Figure S4** Kaplan-Meier curves for non-fatal stroke by diabetes status (A), PNI level (B) and both (C) 14](file:///C:\Users\Tianyu%20Li\Documents\Fuwai\5%20项目\2021-2022\Research_PNI%20(2022.6)\4%20Publication\CD\Additional%20file.docx#_Toc119793882)

[**Additional file 1 Figure S5** Kaplan-Meier curves for unplanned revascularization by diabetes status (A), PNI level (B) and both (C) 15](file:///C:\Users\Tianyu%20Li\Documents\Fuwai\5%20项目\2021-2022\Research_PNI%20(2022.6)\4%20Publication\CD\Additional%20file.docx#_Toc119793883)

[**Additional file 1 Figure S6** Kaplan-Meier curves for MACCE by diabetes status (A), PNI level (B) and both (C) 16](file:///C:\Users\Tianyu%20Li\Documents\Fuwai\5%20项目\2021-2022\Research_PNI%20(2022.6)\4%20Publication\CD\Additional%20file.docx#_Toc119793884)

[**Additional file 1 Figure S7** Central illustration 18](file:///C:\Users\Tianyu%20Li\Documents\Fuwai\5%20项目\2021-2022\Research_PNI%20(2022.6)\4%20Publication\CD\Additional%20file.docx#_Toc119793885)

**Additional file 1: Table S1** Baseline characteristics stratified by follow-up status

| **Variable** | **Completed follow-up**  **(n=9429)** | **Lost to follow-up**  **(n=834)** | **P** |
| --- | --- | --- | --- |
| **Demographic characteristics** |  |  |  |
| Sex (Women) | 2162 (22.93) | 195 (23.38) | 0.766 |
| Age, years | 59 [51, 66] | 58 [50, 65] | 0.051 |
| ≥65 | 2623 (27.82) | 225 (26.98) | 0.604 |
| BMI, kg/m^2^ | 25.91 [23.88, 27.76] | 25.95 [23.83, 27.83] | 0.773 |
| ≥25 | 5742 (60.90) | 501 (60.07) | 0.640 |
| Current smoking | 5363 (56.88) | 482 (57.79) | 0.609 |
| **Clinical characteristics** |  |  |  |
| Clinical presentation |  |  | 0.134 |
| ACS | 5583 (59.21) | 516 (61.87) |  |
| CCS | 3846 (40.79) | 318 (38.13) |  |
| Hypertension | 6576 (69.74) | 577 (69.18) | 0.737 |
| Dyslipidemia | 7121 (75.52) | 630 (75.54) | 0.991 |
| Diabetes | 3956 (41.96) | 345 (41.37) | 0.741 |
| Peripheral artery disease | 252 (2.67) | 25 (3.00) | 0.579 |
| COPD | 220 (2.33) | 18 (2.16) | 0.748 |
| Previous revascularization | 2468 (26.17) | 190 (22.78) | 0.032 |
| Previous MI | 1826 (19.37) | 152 (18.23) | 0.424 |
| Previous stroke | 990 (10.50) | 90 (10.79) | 0.792 |
| Medication at admission |  |  |  |
| Aspirin | 9315 (98.79) | 825 (98.92) | 0.741 |
| Clopidogrel | 9412 (99.82) | 830 (99.52) | 0.184 |
| Statins | 9051 (95.99) | 799 (95.80) | 0.791 |
| β-blockers | 8493 (90.07) | 766 (91.85) | 0.098 |
| ACEIs/ARBs | 4929 (52.27) | 421 (50.48) | 0.320 |
| **Preprocedural laboratory tests** |  |  |  |
| ALC, 10^9^/L | 1.87 [1.51, 2.30] | 1.87 [1.50, 2.33] | 0.956 |
| Serum albumin, g/L | 42.70 [39.90, 45.90] | 42.70 [39.70, 45.77] | 0.537 |
| Preprocedural PNI | 52.60 [49.00, 56.15] | 52.50 [48.86, 56.25] | 0.744 |
| Fasting blood glucose, mmol/L | 5.48 [4.93, 6.63] | 5.48 [4.91, 6.56] | 0.992 |
| HbA1c, % | 6.2 [5.8, 6.9] | 6.2 [5.8, 7.0] | 0.408 |
| hs-CRP, mg/L | 1.73 [0.80, 3.95] | 1.60 [0.80, 3.59] | 0.149 |
| eGFR, ml/min/1.73m^2^ | 118.11 [102.63, 133.24] | 119.10 [102.60, 137.34] | 0.063 |
| ≤60 | 92 (0.98) | 11 (1.32) | 0.340 |
| LVEF, % | 64 [60, 67] | 64 [60, 68] | 0.336 |
| <40 | 102 (1.08) | 11 (1.32) | 0.529 |
| **Angiographic characteristics** | |  |  |
| LM/TVD | 412 (4.37) | 34 (4.08) | 0.691 |
| SYNTAX score | 10 [6, 17] | 10 [6, 17] | 0.959 |
| SYNTAX category |  |  | 0.315 |
| ≤22 | 8367 (88.74) | 744 (89.21) |  |
| 22-32 | 893 (9.47) | 81 (9.71) |  |
| ≥33 | 169 (1.79) | 9 (1.08) |  |
| DES implantation | 8950 (94.92) | 789 (94.60) | 0.691 |

Values are presented as number (%) or median [interquartile range].

Abbreviations: ACEI, angiotensin-converting enzyme inhibitor; ACS, acute coronary syndrome; ALC, absolute lymphocyte counts; ARB, angiotensin-receptor blocker; BMI, body mass index; CCS, chronic coronary syndrome; COPD, chronic obstructive pulmonary disease; DES, drug-eluting stent; eGFR, estimated glomerular filtration rate; HbA1c, glycated hemoglobin; hs-CRP, high-sensitivity C-reactive protein; LM/TVD, left main or three-vessel disease; LVEF, left ventricular ejection fraction; MI, myocardial infarction; PNI, prognostic nutritional index; SYNTAX, synergy between percutaneous coronary intervention with Taxus and cardiac surgery.

**Additional file 1: Table S2** Correlation between preprocedural PNI and glycemic measures in all participants and stratified by DM status

| **Variables** | **Pearson correlation**  **coefficients** | **95% Confidence interval** | **p** |
| --- | --- | --- | --- |
| All participants |  |  |  |
| Fasting blood glucose, mmol/L | 0.071 | 0.051, 0.091 | <0.001 |
| HbA1c, % | 0.029 | 0.009, 0.049 | 0.005 |
| DM |  |  |  |
| fasting blood glucose, mmol/L | 0.042 | 0.010, 0.073 | 0.009 |
| HbA1c, % | 0.011 | -0.020, 0.042 | 0.486 |
| Non-DM |  |  |  |
| fasting blood glucose, mmol/L | 0.184 | 0.158, 0.209 | <0.001 |
| HbA1c, % | 0.003 | -0.023, 0.030 | 0.810 |

Abbreviations: DM, diabetes; HbA1c, glycated hemoglobin; PNI, prognostic nutritional index.

**Additional file 1: Table S3** Univariate and multivariate Cox proportional-hazard regression analysis for all-cause death

| **Variables** | **Univariate analysis** |  | **Multivariate analysis** |  |
| --- | --- | --- | --- | --- |
|  | **HR (95% CI)** | **p** | **HR (95% CI)** | **p** |
| Category |  |  |  |  |
| Non-DM/H-PNI | Reference | - | Reference | - |
| Non-DM/L-PNI | 1.53 (1.11, 2.10) | 0.009 | 1.44 (1.05, 1.98) | 0.026 |
| DM/H-PNI | 1.26 (0.97, 1.62) | 0.080 | 1.16 (0.90, 1.51) | 0.248 |
| DM/L-PNI | 3.30 (2.47, 4.39) | <0.001 | 2.65 (1.97, 3.56) | <0.001 |
| Sex, men as reference | 1.18 (0.94, 1.50) | 0.158 | 1.18 (0.89, 1.56) | 0.263 |
| Age, per 1 year | 1.08 (1.06, 1.09) | <0.001 | 1.07 (1.06, 1.08) | <0.001 |
| Age ≥65 | 3.33 (2.71, 4.09) | <0.001 |  |  |
| BMI, per 1 kg/m^2^ | 0.96 (0.93, 0.99) | 0.013 |  |  |
| BMI ≥25 | 0.89 (0.72, 1.10) | 0.271 |  |  |
| Current smoking | 1.02 (0.83, 1.26) | 0.847 |  |  |
| ACS, CCS as reference | 1.06 (0.86, 1.30) | 0.603 |  |  |
| Hypertension | 1.70 (1.32, 2.19) | <0.001 | 1.56 (1.21, 2.02) | 0.001 |
| Dyslipidemia | 0.89 (0.71, 1.13) | 0.347 |  |  |
| Peripheral artery disease | 1.69 (1.02, 2.78) | 0.041 |  |  |
| COPD | 3.06 (2.02, 4.62) | <0.001 | 2.68 (1.76, 4.07) | <0.001 |
| Previous revascularization | 1.65 (1.34, 2.04) | <0.001 | 1.46 (1.17, 1.83) | 0.001 |
| Previous MI | 1.41 (1.11, 1.78) | 0.005 | 1.12 (0.87, 1.45) | 0.389 |
| Previous stroke | 1.40 (1.04, 1.88) | 0.027 | 1.14 (0.84, 1.54) | 0.392 |
| Aspirin at admission | 0.62 (0.29, 1.30) | 0.206 |  |  |
| Clopidogrel at admission | 0.65 (0.09, 4.59) | 0.662 |  |  |
| Statins at admission | 0.97 (0.58, 1.63) | 0.922 |  |  |
| β-blockers at admission | 0.83 (0.60, 1.14) | 0.241 |  |  |
| ACEIs/ARBs at admission | 1.15 (0.94, 1.41) | 0.183 |  |  |
| hs-CRP, per 1 mg/L | 1.06 (1.03, 1.08) | <0.001 | 1.04 (1.01, 1.06) | 0.003 |
| eGFR, per 1 ml/min/1.73m^2^ | 0.99 (0.98, 0.99) | <0.001 |  |  |
| eGFR ≤60 | 5.09 (3.08, 8.39) | <0.001 | 2.67 (1.58, 4.52) | <0.001 |
| LVEF, per 1 % | 0.96 (0.95, 0.97) | <0.001 |  |  |
| LVEF <40 | 4.00 (2.34, 6.82) | <0.001 | 2.62 (1.51, 4.54) | 0.001 |
| LM/TVD | 1.14 (0.71, 1.83) | 0.594 |  |  |
| SYNTAX score, per 1-point | 1.00 (0.99, 1.02) | 0.582 |  |  |
| SYNTAX category |  |  |  |  |
| ≤22 | Reference | - |  |  |
| 22-32 | 1.30 (0.95, 1.79) | 0.103 |  |  |
| ≥33 | 1.62 (0.86, 3.04) | 0.133 |  |  |
| DES implantation | 0.69 (0.46, 1.02) | 0.064 |  |  |

Preprocedural PNI was categorized by the optimal cut-off value for all-cause death of 48.49.

Abbreviations: ACEI, angiotensin-converting enzyme inhibitor; ACS, acute coronary syndrome; ARB, angiotensin-receptor blocker; BMI, body mass index; CCS, chronic coronary syndrome; CI, confidence interval; COPD, chronic obstructive pulmonary disease; DES, drug-eluting stent; DM, diabetes; eGFR, estimated glomerular filtration rate; H-, high-; HbA1c, glycated hemoglobin; HR, hazard ratio; hs-CRP, high-sensitivity C-reactive protein; L-, low-; LM/TVD, left main or three-vessel disease; LVEF, left ventricular ejection fraction; MI, myocardial infarction; PNI, prognostic nutritional index; SYNTAX, synergy between percutaneous coronary intervention with Taxus and cardiac surgery.**Additional file 1: Table S4** Associations of DM status and PNI level with clinical outcomes after inverse probability of treatment weighting

| **Outcome** | **Hazard ratio (95% Confidence interval)** | **p** |
| --- | --- | --- |
| **All-cause death** | - | - |
| Non-DM/H-PNI | Reference | - |
| Non-DM/L-PNI | 1.47 (1.00, 2.17) | 0.050 |
| DM/H-PNI | 0.93 (0.67, 1.30) | 0.697 |
| DM/L-PNI | 2.09 (1.38, 3.17) | 0.001 |
| p for trend | 0.127 |  |
| **Cardiac death** | - | - |
| Non-DM/H-PNI | Reference | - |
| Non-DM/L-PNI | 1.26 (0.93, 1.71) | 0.167 |
| DM/H-PNI | 0.89 (0.70, 1.15) | 0.412 |
| DM/L-PNI | 1.89 (1.37, 2.60) | <0.001 |
| p for trend | 0.139 | - |
| **Non-fatal MI** | - | - |
| Non-DM/H-PNI | Reference | - |
| Non-DM/L-PNI | 0.86 (0.65, 1.12) | 0.278 |
| DM/H-PNI | 1.04 (0.86, 1.25) | 0.699 |
| DM/L-PNI | 1.04 (0.76, 1.42) | 0.838 |
| p for trend | 0.686 |  |
| **Non-fatal stroke** | - | - |
| Non-DM/H-PNI | Reference | - |
| Non-DM/L-PNI | 1.57 (1.15, 2.14) | 0.005 |
| DM/H-PNI | 1.28 (1.00, 1.64) | 0.050 |
| DM/L-PNI | 1.53 (1.04, 2.23) | 0.036 |
| p for trend | 0.018 | - |
| **Unplanned revascularization** | - | - |
| Non-DM/H-PNI | Reference | - |
| Non-DM/L-PNI | 0.88 (0.74, 1.05) | 0.173 |
| DM/H-PNI | 1.21 (1.07, 1.36) | 0.003 |
| DM/L-PNI | 1.09 (0.89, 1.34) | 0.427 |
| p for trend | <0.001 | - |
| **MACCE** | - | - |
| Non-DM/H-PNI | Reference | - |
| Non-DM/L-PNI | 1.01 (0.88, 1.15) | 0.927 |
| DM/H-PNI | 1.15 (1.04, 1.26) | 0.008 |
| DM/L-PNI | 1.34 (1.15, 1.56) | <0.001 |
| p for trend | <0.001 | - |

Preprocedural PNI was categorized by the optimal cut-off value for all-cause death of 48.49.

The propensity score was generated by logistic regression analysis with sex, age, body mass index, current smoking, acute coronary syndrome, hypertension, dyslipidemia, chronic obstructive pulmonary disease, peripheral artery disease, previous revascularization, previous myocardial infarction, previous stroke, high-sensitivity C-reactive protein, estimated glomerular filtration rate, left ventricular ejection fraction, left main or three-vessel disease, and synergy between percutaneous coronary intervention with Taxus and cardiac surgery category.

CI, confidence interval; DM, diabetes; H-, high; HR, hazard ratio; L-, low; PNI, prognostic nutritional index; pys, person years; MACCE, major adverse cardiovascular and cerebrovascular events; MI, myocardial infarction.

**Additional file 1: Table S4** Baseline characteristics after inverse probability of treatment weighting

| **Variable** | **Non-DM/H-PNI** | **Non-DM/L-PNI** | **DM/H-PNI** | **DM/L-PNI** | **p** |
| --- | --- | --- | --- | --- | --- |
| Demographic characteristics | |  |  |  |  |
| Sex (Women) | 22.52 | 25.52 | 22.30 | 23.27 | 0.1134 |
| Age, years | 58.14±10.32 | 62.49±9.97 | 56.93±9.66 | 61.20±9.50 | <0.001 |
| ≥65 | 27.16 | 42.99 | 21.91 | 35.95 | <0.001 |
| BMI, kg/m^2^ | 26.03±3.21 | 24.96±3.13 | 26.28±3.05 | 25.48±3.15 | <0.001 |
| ≥25 | 61.93 | 49.53 | 65.00 | 53.67 | <0.001 |
| Current smoking | 57.27 | 53.39 | 57.77 | 55.40 | 0.043 |
| Clinical characteristics |  |  |  |  |  |
| Clinical presentation |  |  |  |  | <0.001 |
| ACS | 58.71 | 68.26 | 69.95 | 71.30 |  |
| CCS | 41.29 | 33.67 | 30.05 | 28.70 |  |
| Hypertension | 70.19 | 68.26 | 69.95 | 71.30 | 0.481 |
| Dyslipidemia | 76.39 | 68.83 | 78.19 | 72.92 | <0.001 |
| Peripheral artery disease | 2.82 | 2.19 | 2.65 | 3.31 | 0.460 |
| COPD | 2.22 | 3.57 | 1.86 | 2.96 | 0.004 |
| Previous revascularization | 26.49 | 25.30 | 25.97 | 25.53 | 0.825 |
| Previous MI | 19.74 | 18.57 | 19.12 | 19.92 | 0.761 |
| Previous stroke | 11.14 | 10.84 | 9.72 | 12.53 | 0.083 |
| Medication at admission |  |  |  |  |  |
| Aspirin | 98.93 | 97.88 | 98.89 | 98.66 | 0.225 |
| Clopidogrel | 98.75 | 97.87 | 98.46 | 98.79 | 0.128 |
| Statins | 96.11 | 97.26 | 95.51 | 94.66 | 0.014 |
| β-blockers | 89.26 | 88.72 | 91.51 | 89.84 | 0.005 |
| ACEIs/ARBs | 50.57 | 49.66 | 55.51 | 56.43 | <0.001 |
| Preprocedural laboratory tests | |  |  |  |  |
| ALC, 10^9^/L | 2.05±0.60 | 1.46±0.38 | 2.13±0.67 | 1.48±0.41 | <0.001 |
| Serum albumin, g/L | 44.02±3.44 | 38.38±2.37 | 44.20±3.49 | 38.44±2.50 | <0.001 |
| PNI | 54.28±3.88 | 45.70±2.27 | 54.86±4.28 | 45.85±2.29 | <0.001 |
| hs-CRP, mg/L | 3.14±3.74 | 3.88±4.43 | 2.79±3.25 | 3.75±4.42 | <0.001 |
| Fasting blood glucose, mmol/L | 5.22±0.59 | 5.03±0.56 | 7.54±2.39 | 7.25±2.62 | <0.001 |
| Glycated hemoglobin, % | 5.94±0.34 | 5.93±0.35 | 7.56±1.30 | 7.57±1.53 | <0.001 |
| eGFR, ml/min/1.73m^2^ | 117.30±23.69 | 115.98±25.44 | 120.23±24.63 | 119.21±27.98 | <0.001 |
| ≤60 | 1.20 | 1.15 | 0.64 | 1.60 | 0.034 |
| LVEF, % | 63±7 | 62±8 | 63±7 | 62±8 | <0.001 |
| <40 | 1.10 | 1.47 | 0.80 | 1.84 | 0.046 |
| Angiographic characteristics | |  |  |  |  |
| LM/TVD | 4.55 | 4.46 | 4.15 | 5.06 | 0.702 |
| SYNTAX score | 11±8 | 12±8 | 12±8 | 12±9 | 0.078 |
| SYNTAX category |  |  |  |  | 0.730 |
| ≤22 | 88.27 | 88.90 | 89.08 | 86.95 |  |
| 22-32 | 9.93 | 9.16 | 9.22 | 10.95 |  |
| ≥33 | 1.80 | 1.94 | 1.70 | 2.11 |  |
| DES implantation | 95.40 | 94.30 | 95.08 | 94.62 | 0.408 |

Preprocedural PNI was categorized by the optimal cut-off value for all-cause death of 48.49.

Values are presented as percentage or mean ± standard deviation.

The propensity score was generated by logistic regression analysis with sex, age, BMI, current smoking, ACS, hypertension, dyslipidemia, COPD, peripheral artery disease, previous revascularization, previous myocardial infarction, previous stroke, hsCRP, eGFR, lVEF, LM/TVD, and SYNTAX category.

ACEI, angiotensin-converting enzyme inhibitor; ACS, acute coronary syndrome; ALC, absolute lymphocyte counts; ARB, angiotensin-receptor blocker; BMI, body mass index; CCS, chronic coronary syndrome; COPD, chronic obstructive pulmonary disease; DES, drug-eluting stent; DM, diabetes; eGFR, estimated glomerular filtration rate; H-, high; hs-CRP, high-sensitivity C-reactive protein; L-, low; LM/TVD, left main or three-vessel disease; LVEF, left ventricular ejection fraction; MI, myocardial infarction; PNI, prognostic nutritional index; SYNTAX, synergy between percutaneous coronary intervention with Taxus and cardiac surgery.

**Additional file 1: Table S6** Subgroup analysis for all-cause death

|  | **Events/Total** | **Event rate per 1000 pys** | **Crude HR (95% CI)** | **p** | **Adjusted HR (95% CI)** | **p** | **p_interaction_** |
| --- | --- | --- | --- | --- | --- | --- | --- |
| **Age <65** | 163/6806 | 4.85 |  |  |  |  | 0.101 |
| Non-DM/H-PNI | 66/3345 | 3.96 | Reference |  | Reference |  |  |
| Non-DM/L-PNI | 21/728 | 5.82 | 1.47 (0.90, 2.40) | 0.124 | 1.43 (0.88, 2.34) | 0.152 |  |
| DM/H-PNI | 61/2295 | 5.36 | 1.35 (0.96, 1.92) | 0.089 | 1.24 (0.88, 1.76) | 0.223 |  |
| DM/L-PNI | 15/438 | 6.93 | 1.75 (1.00, 3.07) | 0.050 | 1.42 (0.80, 2.51) | 0.228 |  |
| p for trend |  |  | 0.027 |  | 0.142 |  |  |
| **Age ≥65** | 203/2623 | 16.00 |  |  |  |  |  |
| Non-DM/H-PNI | 56/872 | 13.15 | Reference |  | Reference |  |  |
| Non-DM/L-PNI | 34/528 | 13.19 | 1.00 (0.66, 1.54) | 0.986 | 0.99 (0.65, 1.52) | 0.966 |  |
| DM/H-PNI | 52/835 | 12.85 | 0.98 (0.67, 1.43) | 0.914 | 0.97 (0.66, 1.42) | 0.880 |  |
| DM/L-PNI | 61/388 | 33.85 | 2.59 (1.80, 3.72) | <0.001 | 2.38 (1.65, 3.44) | <0.001 |  |
| p for trend |  |  | <0.001 |  | <0.001 |  |  |
| **Women** | 95/2162 | 8.94 |  |  |  |  | 0.627 |
| Non-DM/H-PNI | 27/835 | 6.53 | Reference |  | Reference |  |  |
| Non-DM/L-PNI | 16/318 | 10.22 | 1.57 (0.84, 2.91) | 0.155 | 1.54 (0.83, 2.86) | 0.173 |  |
| DM/H-PNI | 27/784 | 7.00 | 1.07 (0.63, 1.83) | 0.793 | 1.01 (0.59, 1.74) | 0.960 |  |
| DM/L-PNI | 25/225 | 24.28 | 3.59 (2.08, 6.18) | <0.001 | 3.12 (1.78, 5.45) | <0.001 |  |
| p for trend |  |  | 0.001 |  | 0.007 |  |  |
| **Men** | 271/7267 | 7.55 |  |  |  |  |  |
| Non-DM/H-PNI | 95/3382 | 5.66 | Reference |  | Reference |  |  |
| Non-DM/L-PNI | 39/938 | 8.44 | 1.49 (1.03, 2.17) | 0.035 | 1.38 (0.95, 2.01) | 0.089 |  |
| DM/H-PNI | 86/2346 | 7.43 | 1.31 (0.98, 1.76) | 0.066 | 1.22 (0.91, 1.63) | 0.186 |  |
| DM/L-PNI | 51/601 | 17.61 | 3.12 (2.22, 4.38) | <0.001 | 2.44 (1.72, 3.46) | <0.001 |  |
| p for trend |  |  | <0.001 |  | <0.001 |  |  |
| **BMI <25** | 153/3687 | 8.43 |  |  |  |  | 0.237 |
| Non-DM/H-PNI | 50/1662 | 6.08 | Reference |  | Reference |  |  |
| Non-DM/L-PNI | 35/637 | 11.19 | 1.84 (1.19, 2.83) | 0.006 | 1.78 (1.15, 2.75) | 0.009 |  |
| DM/H-PNI | 35/1042 | 6.84 | 1.13 (0.73, 1.74) | 0.588 | 1.06 (0.69, 1.64) | 0.793 |  |
| DM/L-PNI | 33/346 | 5.49 | 3.25 (2.09, 5.04) | <0.001 | 2.83 (1.81, 4.42) | <0.001 |  |
| p for trend |  |  | <0.001 |  | 0.001 |  |  |
| **BMI ≥25** | 213/5742 | 7.51 |  |  |  |  |  |
| Non-DM/H-PNI | 72/2555 | 5.67 | Reference |  | Reference |  |  |
| Non-DM/L-PNI | 20/619 | 6.54 | 1.16 (0.70, 1.90) | 0.568 | 1.42 (1.03, 1.96) | 0.032 |  |
| DM/H-PNI | 78/2088 | 7.56 | 1.33 (0.97, 1.84) | 0.077 | 1.17 (0.91, 1.52) | 0.226 |  |
| DM/L-PNI | 43/480 | 18.78 | 3.32 (2.28, 4.84) | <0.001 | 2.64 (1.97, 3.55) | <0.001 |  |
| p for trend |  |  | <0.001 |  | 0.002 |  |  |
| **ACS** | 221/5583 | 8.05 |  |  |  |  | 0.321 |
| Non-DM/H-PNI | 69/2473 | 5.63 | Reference |  | Reference |  |  |
| Non-DM/L-PNI | 42/832 | 10.29 | 1.83 (1.25, 2.69) | 0.002 | 1.71 (1.16, 2.51) | 0.006 |  |
| DM/H-PNI | 60/1721 | 7.10 | 1.26 (0.89, 1.78) | 0.187 | 1.15 (0.81, 1.63) | 0.433 |  |
| DM/L-PNI | 50/557 | 18.73 | 3.33 (2.32, 4.80) | <0.001 | 2.67 (1.83, 3.88) | <0.001 |  |
| p for trend |  |  | <0.001 |  | <0.001 |  |  |
| **CCS** | 145/3846 | 7.62 |  |  |  |  |  |
| Non-DM/H-PNI | 53/1744 | 6.11 | Reference |  | Reference |  |  |
| Non-DM/L-PNI | 13/424 | 6.18 | 1.01 (0.55, 1.85) | 0.973 | 0.94 (0.51, 1.73) | 0.846 |  |
| DM/H-PNI | 53/1409 | 7.60 | 1.24 (0.85, 1.82) | 0.263 | 1.19 (0.81, 1.74) | 0.374 |  |
| DM/L-PNI | 26/269 | 20.03 | 3.29 (2.06, 5.26) | <0.001 | 2.67 (1.65, 4.32) | <0.001 |  |
| p for trend |  |  | <0.001 |  | 0.003 |  |  |

Preprocedural PNI was categorized by the optimal cut-off value for all-cause death of 48.49.

Adjusted for sex, age, hypertension, chronic obstructive pulmonary disease, previous revascularization, previous myocardial infarction, previous stroke, high-sensitivity C-reactive protein, estimated glomerular filtration rate, and left ventricular ejection fraction.

Abbreviations: ACS, acute coronary syndrome; BMI, body mass index; CCS, chronic coronary syndrome; CI, confidence interval; DM, diabetes; H-, high-; HR, hazard ratio; L-, low-; PNI, prognostic nutritional index; pys, person years.

**Additional file 1: Table S7** Sensitivity analysis for all-cause death

|  | **Events/Total** | **Event rate**  **per 1000 pys** | **Crude HR**  **(95% CI)** | **p** | **Adjusted HR**  **(95% CI)** | **p** |
| --- | --- | --- | --- | --- | --- | --- |
| **Dichotomous PNI^a^** | 366/9429 | 7.87 |  |  |  |  |
| Non-DM/H-PNI | 76/2701 | 5.67 | Reference |  | Reference |  |
| Non-DM/L-PNI | 101/2772 | 7.38 | 1.30 (0.97, 1.75) | 0.082 | 1.23 (0.92, 1.66) | 0.168 |
| DM/H-PNI | 64/2023 | 6.40 | 1.13 (0.81, 1.58) | 0.469 | 1.04 (0.75, 1.46) | 0.810 |
| DM/L-PNI | 125/1933 | 13.30 | 2.35 (1.77, 3.13) | <0.001 | 1.99 (1.49, 2.66) | <0.001 |
| p for trend |  |  | <0.001 |  | <0.001 |  |
| **Continuous PNI^b^** |  |  |  |  |  |  |
| All participants | 366/9429 | 7.87 | 0.93 (0.91, 0.95) | <0.001 | 0.94 (0.92, 0.96) | <0.001 |
| DM | 189/3956 | 9.74 | 0.91 (0.88, 0.93) | <0.001 | 0.92 (0.89, 0.95) | <0.001 |
| Non-DM | 178/5473 | 6.57 | 0.96 (0.93, 0.98) | 0.002 | 0.96 (0.93, 0.99) | 0.006 |
| **Postprocedural PNI^c^** | 327/8438 | 7.86 |  |  |  |  |
| Non-DM/H-PNI | 50/2235 | 4.49 | Reference |  | Reference |  |
| Non-DM/L-PNI | 108/2663 | 8.22 | 1.83 (1.31, 2.56) | <0.001 | 1.76 (1.25, 2.46) | 0.001 |
| DM/H-PNI | 57/1754 | 6.56 | 1.46 (1.00, 2.14) | 0.050 | 1.35 (0.92, 1.98) | 0.122 |
| DM/L-PNI | 112/1786 | 12.91 | 2.88 (2.07, 4.03) | <0.001 | 2.43 (1.73, 3.42) | <0.001 |
| p for trend |  |  | <0.001 |  | <0.001 |  |
| **ΔPNI^d^** |  |  |  |  |  |  |
| All participants | 327/8438 | 7.86 | 1.00 (0.97, 1.02) | 0.645 | 0.99 (0.97, 1.01) | 0.504 |
| DM | 169/3540 | 9.73 | 1.01 (0.98, 1.04) | 0.409 | 1.01 (0.98, 1.04) | 0.593 |
| Non-DM | 158/4898 | 6.51 | 0.98 (0.95, 1.01) | 0.122 | 0.97 (0.95, 1.01) | 0.108 |
| **GLIM^e^** | 366/9429 | 7.87 |  |  |  |  |
| Non-DM/well-nourished | 152/5211 | 5.88 | Reference |  | Reference |  |
| Non-DM/malnourished | 25/262 | 20.05 | 3.42 (2.24, 5.22) | <0.001 | 2.98 (1.94, 4.58) | <0.001 |
| DM/well-nourished | 162/3747 | 8.79 | 1.50 (1.20, 1.87) | <0.001 | 1.35 (1.08, 1.69) | 0.009 |
| DM/malnourished | 27/209 | 28.01 | 4.80 (3.19, 7.23) | <0.001 | 4.25 (2.79, 6.48) | <0.001 |
| p for trend |  |  | <0.001 |  | <0.001 |  |

^a^ Preprocedural PNI grouped by the median.

^b^ Preprocedural PNI analyzed as a continuous variable.

^c^ Postprocedural PNI grouped by 48.49.

^d^ A continuous variable calculated as postprocedural PNI minus preprocedural PNI.

^e^ Malnutrition defined based on the GLIM criteria.

Adjusted for sex, age, hypertension, chronic obstructive pulmonary disease, previous revascularization, previous myocardial infarction, previous stroke, high-sensitivity C-reactive protein, estimated glomerular filtration rate, and left ventricular ejection fraction.

Abbreviations: CI, confidence interval; DM, diabetes; GLIM, global leadership initiative on malnutrition; H-, high; HR, hazard ratio; L-, low; PNI, prognostic nutritional index; pys, person years.

**
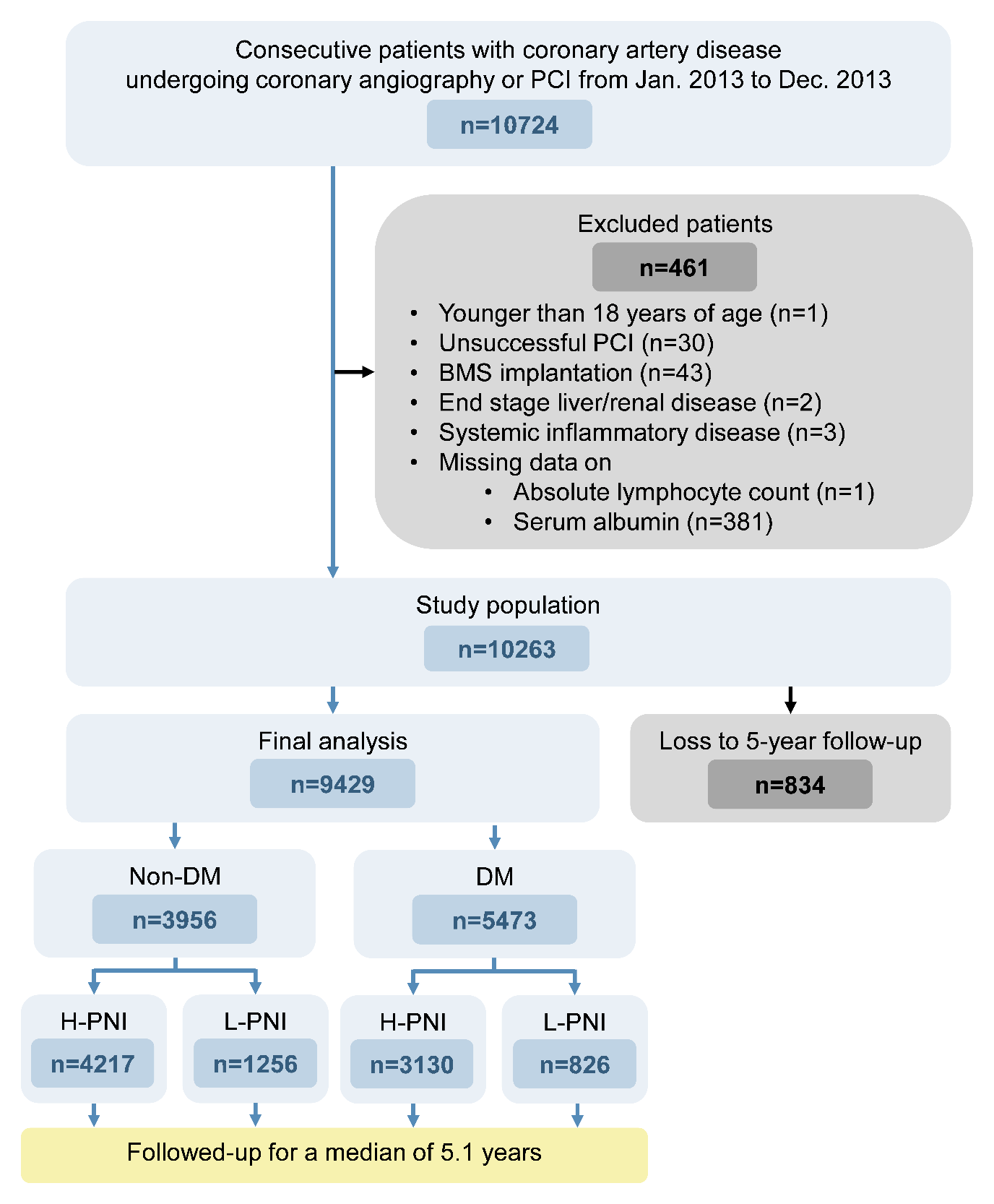
**Preprocedural PNI was categorized by the optimal cut-off value for all-cause death of 48.49. Abbreviations: BMS, bare-metal stent; DM, diabetes; H-, high-; L-, low-; PCI, percutaneous coronary intervention; PNI, prognostic nutritional index.

**Additional file 1: Figure S1** The study flowchart


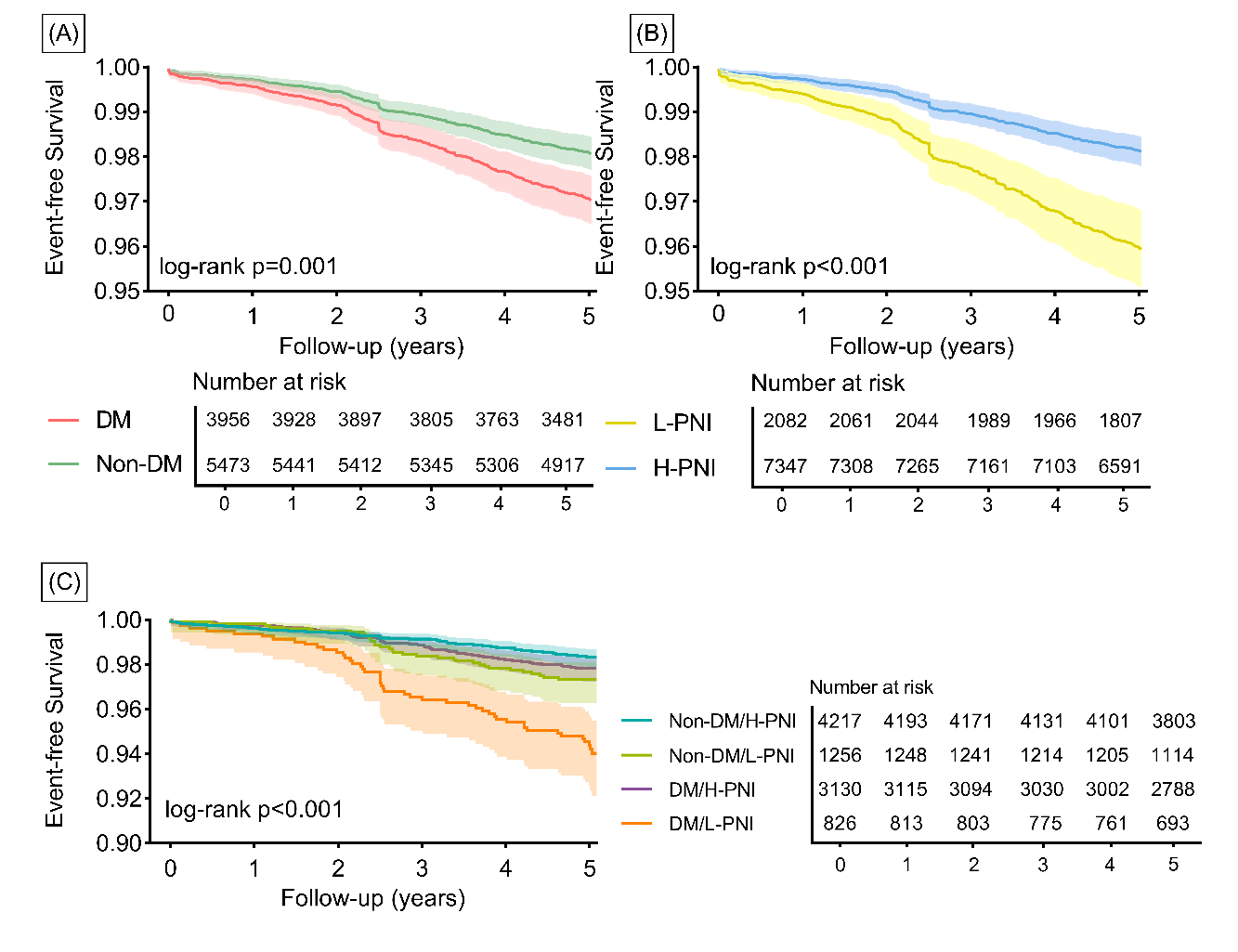
Preprocedural PNI was categorized by the optimal cut-off value for all-cause death of 48.49. Abbreviations: DM, diabetes; PNI, prognostic nutritional index; H-, high-; L-, low-.

**Additional file 1: Figure S2** Kaplan-Meier curves for cardiac death by diabetes status (A), PNI level (B) and both (C)

Preprocedural
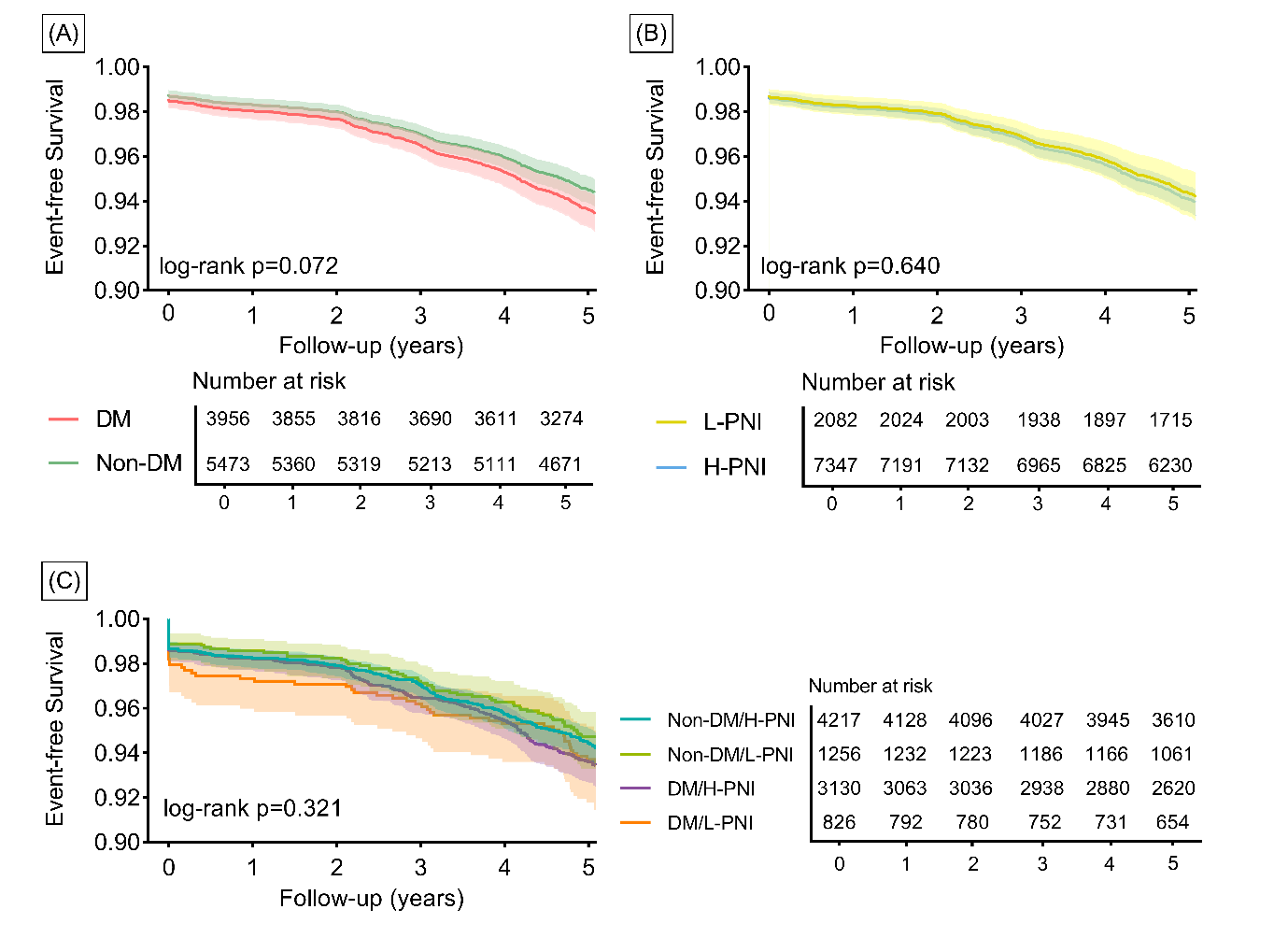
PNI was categorized by the optimal cut-off value for all-cause death of 48.49. Abbreviations: MI, myocardial infarction; DM, diabetes; PNI, prognostic nutritional index; H-, high-; L-, low-

**Additional file 1: Figure S3** Kaplan-Meier curves for non-fatal MI by diabetes status (A), PNI level (B) and both (C)

Preprocedural
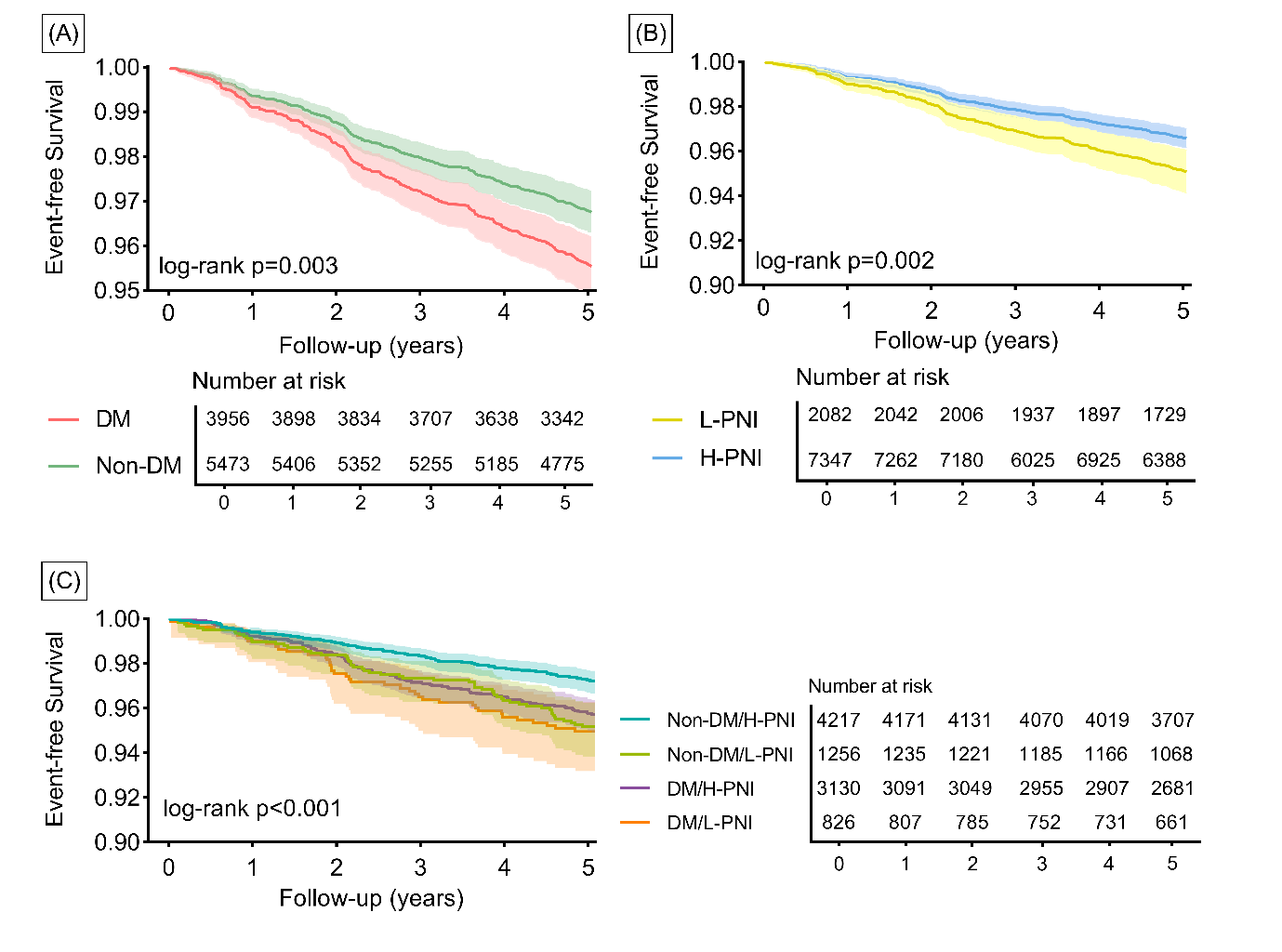
PNI was categorized by the optimal cut-off value for all-cause death of 48.49. Abbreviations: DM, diabetes; PNI, prognostic nutritional index; H-, high-; L-, low-

**Additional file 1: Figure S4** Kaplan-Meier curves for non-fatal stroke by diabetes status (A), PNI level (B) and both (C)

Preprocedural
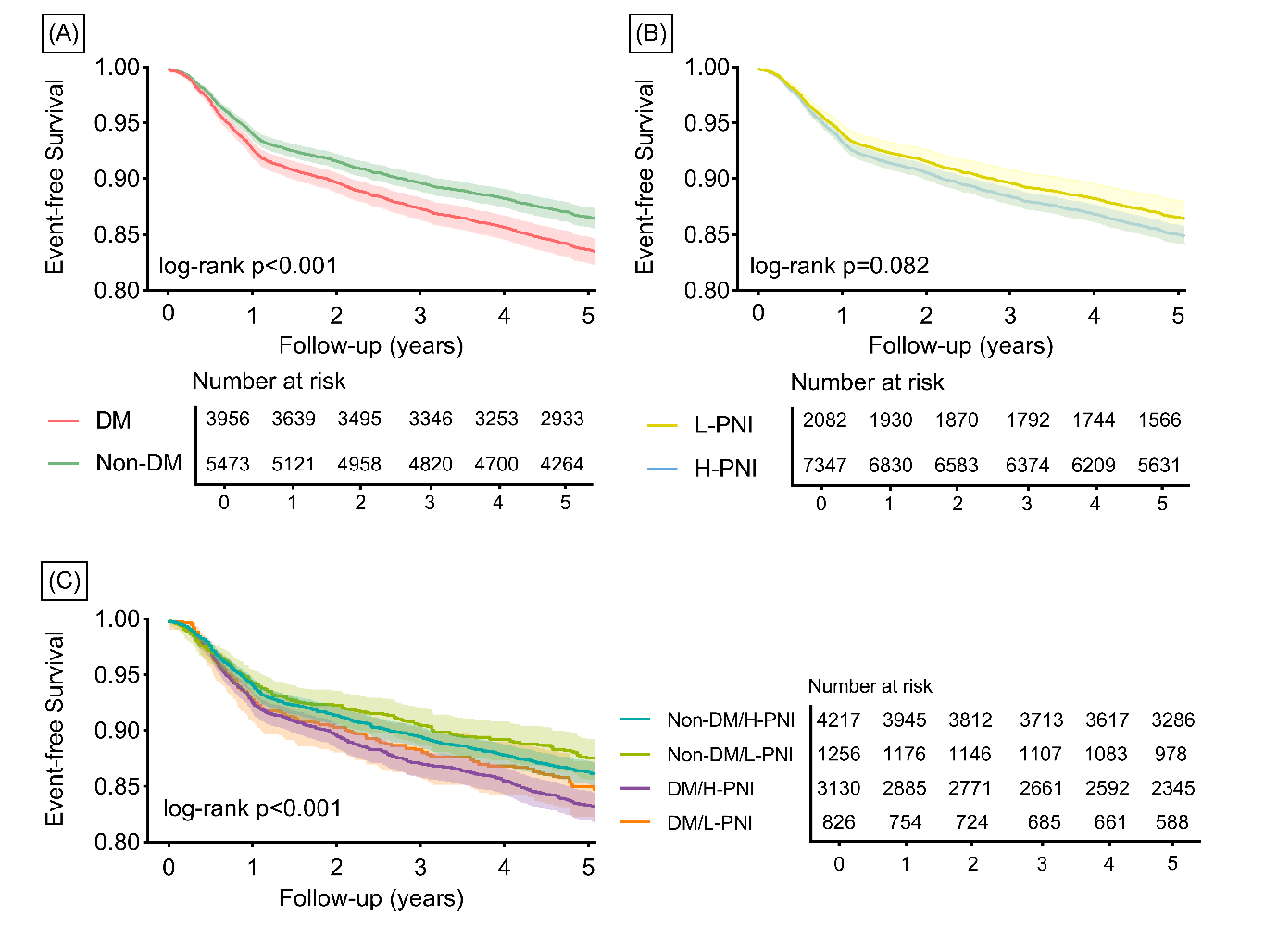
PNI was categorized by the optimal cut-off value for all-cause death of 48.49. Abbreviations: DM, diabetes; PNI, prognostic nutritional index; H-, high-; L-, low-

**Additional file 1: Figure S5** Kaplan-Meier curves for unplanned revascularization by diabetes status (A), PNI level (B) and both (C)

Preprocedural
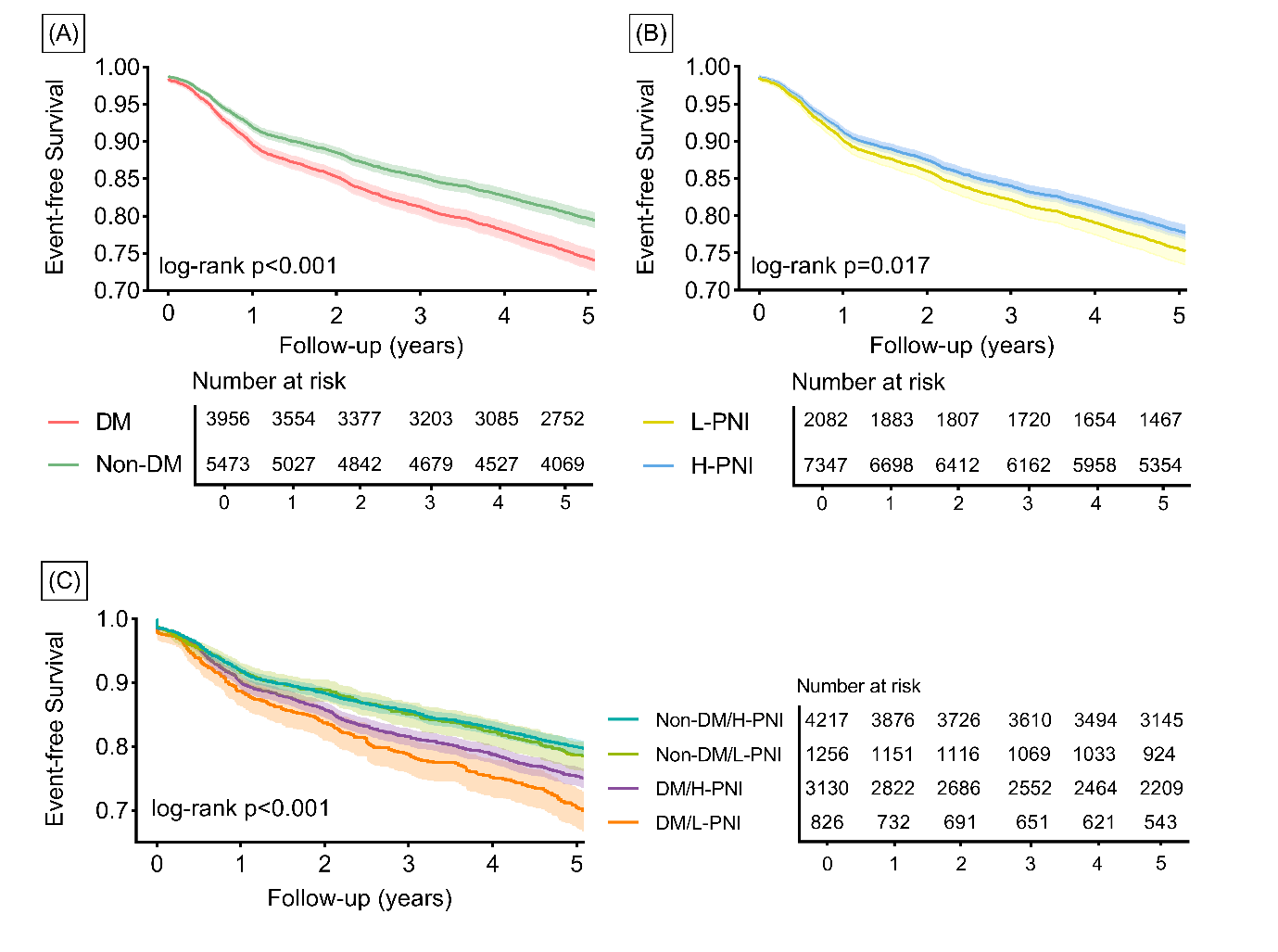
PNI was categorized by the optimal cut-off value for all-cause death of 48.49. Abbreviations: MACCE, major adverse cardiovascular and cerebrovascular events; DM, diabetes; PNI, prognostic nutritional index; H-, high-; L-, low-

**Additional file 1: Figure S6** Kaplan-Meier curves for MACCE by diabetes status (A), PNI level (B) and both (C)


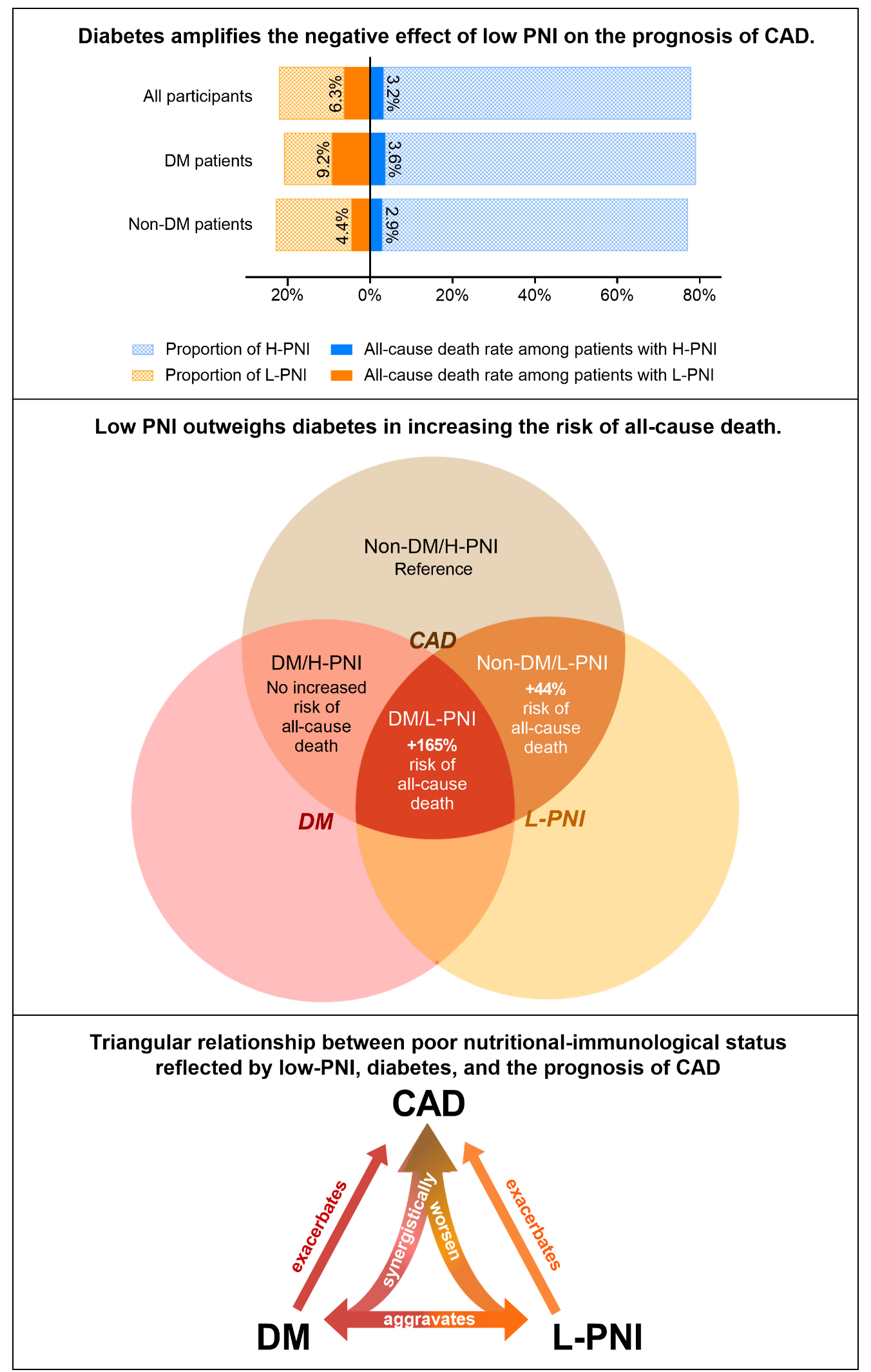


The first panel illustrates that CAD patients with L-PNI had a higher incidence of all-cause death than those with H-PNI, and the difference was more significant in diabetic patients. The second panel illustrates that CAD patients with diabetes and L-PNI experienced the highest risk of all-cause death; L-PNI outweighs diabetes in increasing the risk of all-cause death. The third illustrates a triangular relationship between poor nutritional-immunological status reflected by L-PNI, diabetes, and prognosis of CAD: diabetes and L-PNI exacerbate prognosis of CAD, respectively; diabetes and L-PNI aggravate each other and synergistically worsen the prognosis of CAD. Preprocedural PNI was categorized by the optimal cut-off value for all-cause death of 48.49. CAD, coronary artery disease; DM, diabetes; H-, high-; L-, low-; PNI, prognostic nutritional index.

**Additional file 1: Figure S7** Central illustration
